# Chronic administration of metformin exerts cytostatic and cytotoxic effects via the PP2A-GSK3β-MCL-1 pathway by inhibiting the tmCLIC1 membrane protein in glioblastoma-initiating cells

**DOI:** 10.1186/s13046-025-03577-3

**Published:** 2025-11-24

**Authors:** Francesca Cianci, Ivan Verduci, Riccardo Cazzoli, Gaetano Cannavale, Guido Rey, Marina Veronesi, Beatrice Balboni, Matteo Ranucci, Luca Maria Giovanni Palloni, Federico Ballabio, Noemi Barsotti, Giorgia Ailuno, Alice Balboni, Sara Baldassari, Gabriele Caviglioli, Carlotta Tacconi, Carlo Camilloni, Stefania Girotto, Federica Barbieri, Alessandro Fantin, Andrea Cavalli, Massimo Pasqualetti, Tullio Florio, Saverio Minucci, Michele Mazzanti

**Affiliations:** 1 Department of Bioscience, University of Milan, Via Celoria 26, Milano, 20133 Italy; 2 IEO, Istituto Europeo di Oncologia, Milano, Italy; 3 Structural Biophysics Facility, Istituto Italiano di Tecnologia, Genoa, Italy; 4 D3 PharmaChemistry, Istituto Italiano di Tecnologia, Genoa, Italy; 5 Computational and Chemical Biology, Istituto Italiano di Tecnologia, Genova, Italy; 6 Department of Internal Medicine, Università di Genova, Genova, Italy; 7 IRCCS Ospedale Policlinico San Martino, Genova, Italy; 8 Department of. Pharmacy, Università di Genova, Genova, Italy; 9 Department of Biology, Unit of Cell and Developmental Biology, University of Pisa, Pisa, 56127 Italy; 10 Present address: 3Brain IT SRL, Genova , Italy; 11 Present address: University of New South Wales, Kensington, NSW, Sydney, Australia; 12 Present address: Dept. Pharmacology and Toxicology, University of Zurich, Zurich, Switzerland; 13 Present address: IIT, Genova, Italy; 14 Present address: Vita-Salute San Raffaele University, Milan, Italy; 15 Department of Pathophysiology and Transplantation, University of Milano, Milano, Italy; 16 Department of Experimental Oncology, IEO, European Institute of Oncology IRCCS, Milan, Italy

**Keywords:** Glioblastoma, Metformin, Oxidative phosphorylation, TmCLIC1, Cancer metabolism

## Abstract

**Background:**

One of the main challenges in cancer treatment is addressing the metabolic reprogramming of tumor cells, which require more energy and biomolecules than healthy cells. Cancer cells alter their metabolism by switching between glycolysis and oxidative phosphorylation (OXPHOS). These processes depend on transmembrane proteins that respond to the extracellular environment. Our research identified the transmembrane form of Chloride Intracellular Channel 1 (tmCLIC1) as a marker of malignancy and a potential therapeutic target. tmCLIC1 levels are increased in several solid tumors, supporting cancer growth and progression, whereas they are mostly absent in healthy cells. We confirmed that tmCLIC1 is the specific target of the antidiabetic drug metformin, an OXPHOS inhibitor in cancer cells.

**Methods:**

tmCLIC1 is the primary target of metformin in glioblastoma-initiating cells, as shown by single-channel patch-clamp recordings and NMR experiments. Various patient-derived glioblastoma cells with different genetic backgrounds were used to demonstrate that CLIC1 CRISPR-Cas9 knockout and/or its point mutation at arginine 29 removes metformin’s antitumor effects. Functional assays were used to assess the effects on proliferation, mitochondrial metabolism, and tumor growth in vitro and in vivo, using zebrafish and murine xenograft models.

**Results:**

Metformin inhibits the function of tmCLIC1 through direct and specific binding involving arginine 29 in the tmCLIC1 sequence. Additionally, during hypoglycemia, metformin promotes glioblastoma cell apoptosis by inhibiting the Cancerous Inhibitor of Protein Phosphatase 2 A (CIP2A) and activating the PP2A B56δ subunit. This leads to the dephosphorylation of Glycogen Synthase Kinase 3 Beta (GSK3β), resulting in the breakdown of the pro-survival protein MCL-1 and subsequent cell death. Inhibition of tmCLIC1 is crucial for this metformin-driven antineoplastic effect, mainly through regulating the PP2A-GSK3β-MCL-1 pathway under hypoglycemic conditions. The chronic presence of metformin within the tumors impairs in vivo growth at nanomolar concentrations.

**Conclusions:**

The therapeutic role of metformin to treat brain tumors remains debated. Our findings show that drug delivery is essential, as in vivo, tumor growth decreases at concentrations below 10 nanomolar. We propose that sustained CNS metformin levels may improve tmCLIC1 inhibition, providing a basis for optimizing interactions with metformin or related compounds to enhance therapeutic efficacy.

**Supplementary Information:**

The online version contains supplementary material available at 10.1186/s13046-025-03577-3.

## Background

Glioblastoma (GBM) is the deadliest brain tumor and poses a significant clinical challenge [1]. Despite current standard treatments and advanced strategies [2–6], patient survival rates remain consistently low [7, 8]. Tumor heterogeneity and extensive cell invasiveness make GBM difficult to remove surgically, often leading to recurrence. Scientific consensus indicates that tumor relapse originates from progenitor or stem-like cells called glioblastoma stem cells (GSCs). GSCs have unique tumorigenic potential, including self-renewal and multi-lineage differentiation abilities, which contribute to tumor heterogeneity [9–14]. As a result, GSCs are a promising target for the development of new therapies.

Recently, the anti-cancer properties of metformin have gained attention. When administered to patients with type 2 diabetes, metformin has been associated with a decreased risk of developing various solid cancers and lower cancer-related mortality, including GBM. Additionally, multiple studies on GBM indicate that metformin’s anti-cancer effects specifically target GSCs [15–19].

Despite this evidence, the exact mechanism by which metformin exerts its effects on cancer cells remains unclear. It has been proposed that metformin targets the oxidative phosphorylation (OXPHOS) pathway [20–22]. GSCs can switch between glycolysis and OXPHOS [23–25], and this metabolic flexibility might explain why metformin has seen limited success in clinical trials so far [26].

Chloride Intracellular Channel 1 (CLIC1) is a metamorphic protein primarily located in the cytoplasm under normal conditions. In response to ongoing stress, CLIC1 relocates to the plasma membrane, where it facilitates chloride conductance. Our prior research has demonstrated that the transmembrane form of CLIC1 (tmCLIC1) contributes to GBM progression in vitro and in vivo [27, 28]. Its specific localization and enrichment at the GSC plasma membrane indicate that tmCLIC1 is a promising target for GBM treatment. Notably, treatments with metformin or other agents that inhibit tmCLIC1 activity produce a similar, non-additive reduction in GSC proliferation [29–31]. In this study, we employed a multidisciplinary approach combining molecular, cellular, and in vivo experiments to demonstrate that metformin’s effects on GSC proliferation, survival, and overall GBM growth depend on its inhibition of tmCLIC1.

## Methods

### Reagents

IAA94 (Indanyloxyacetic acid 94; Sigma-Aldrich) was used to inhibit CLIC1 activity specifically. A 50 mM stock solution in absolute ethanol was diluted to 100 µM. Metformin (1,1-dimethylbiguanide hydrochloride; Sigma-Aldrich) was tested as an alternative CLIC1 inhibitor, prepared as a 1 M stock solution in ultrapure water, and applied at final concentrations of 5 or 10 mM. PD0332991 isethionate (Sigma-Aldrich), a CDK4/6 inhibitor, was used at 2.5 µg/ml to synchronize cells in G1 phase. Niclosamide (Sigma-Aldrich), an uncoupler of oxidative phosphorylation, was dissolved at 10 mM in DMSO and used at 4 µM. IACS-010759 (Sigma-Aldrich), a mitochondrial complex I inhibitor, was used at 100 nM from a 10 mM DMSO stock.

### Cell culture

Primary glioblastoma stem cell (GSC) cultures (GBM1, GBM2, GBM3) were derived and maintained per established protocols. GBM1 grew as suspension neurospheres, mechanically dissociated twice weekly. GBM2 and GBM3 adhered to plastic and were occasionally cultured on Geltrex-coated plates.

#### Clic1^−/−^ mutant generation by CRISPR-Cas9 technology

Patient-derived GSCs were transfected with transEDIT lentiviral gRNA plus Cas9 lentiviral expression vector (pCLIP-All-hCMV-ZsGreen V66) according to the manufacturer’s protocol (Transomic). Two plasmids were used, gRNA targeting a *Clic1* coding sequence (TGAGTGCCCCTATACCTGGG) and one targeting the GFP gene as a negative control (NC). Plasmids carry ZsGreen fluorophore as a selection marker.

### Generation of GL261 R29A Knock-In cells using CRISPR-Cas9 technology

The donor double-stranded DNA (dsDNA) template was amplified via PCR from a mutagenized cDNA in which the CLIC1 codon for arginine (AGA) was substituted with alanine (GCC) at position 29 (R29A). The PCR primers used were Forward: GAGCAGCCTGGTAAGGATGG and Reverse: GTGGATGCACGTGCACTGCA, yielding a 225 bp fragment. A total of 1 µg of donor DNA was electroporated into GL261 cells using the Neon Transfection System (ThermoFisher), along with a guide RNA (gRNA) targeting the region upstream of the arginine codon of interest (gRNA sequence: GCAGTGATGGTGCCAAGATT, Integrated DNA Technology) and the Cas9 ribonucleoprotein complex (Integrated DNA Technology). The Cas9-gRNA ribonucleoprotein was pre-assembled in vitro following the manufacturer’s protocol. Successful gene editing was confirmed through sequencing analysis.

### Plasmids

CLIC1wt-pIRES2-EGFP and CLIC1R29A-pIRES2-EGFP were used for in vitro rescue. For in vivo studies, WT and R29A Clic1 were cloned into pCDH vectors for lentiviral transduction.

### OCR/ECAR measurements

Seahorse XF96 Analyzer (Agilent) measured OCR and ECAR in five replicates per condition. Cells were treated with 10 mM metformin during analysis and monitored for up to 120 min.

#### Mitochondrial membrane potential

Cells were treated with 5 mM metformin and stained with JC1 dye. Fluorescence was measured on a BD FACSCelesta™.

### Intracellular ROS and mitochondrial superoxide

Cells were treated ± 3 h with 5 mM metformin, stained with CellROX™ and MitoSOX™, and analyzed via BD FACSCanto™ II. Data were quantified using FlowJo v10.

### Protein extraction and western blotting

Cells were lysed in hot lysis buffer, sonicated, syringed, boiled, and centrifuged. Proteins were quantified by BCA, separated via SDS-PAGE, transferred to nitrocellulose, and probed with primary and secondary antibodies. Detection was by chemiluminescence (ChemiDoc Touch^®^).

### Growth curves

Cells (2 × 10^4^ for GBM1; 7 × 10^3^ for GBM2/3) were plated and counted over time using Trypan Blue exclusion and an automated counter. Triplicates were used per condition.

### 3D Cultures

GBM1 spheroids were formed and transferred after 24–48 h to metformin-containing media (5 mM). Images were captured at 0 and 72 h, and the spheroid area was quantified using ImageJ.

### Fluorescence intensity assay

Cells in suspension (1 × 10^6^ cells/well) were washed three times and incubated in a blocking solution for 30 min on ice. Primary antibody conjugated with Alexa Fluor 555 was added to samples and incubated for 2 h on ice. Samples were washed three times and distributed into a black 96-well plate. Samples were analyzed with an Ensight Multimode Plate Reader (PerkinElmer). Fluorescence intensity values of samples incubated with tmCLIC1omab^®^ (3.5 µg/ml) are proportional to tmCLIC1 and were normalized to their own blank.

### Patch clamp experiments

The voltage step protocol used to isolate current/voltage relationships consisted of 800 ms pulses from − 60 mV to + 60 mV (20 mV voltage steps). The holding potential was set on the cell resting potential. CLIC1-mediated chloride currents were isolated by perfusing IAA94 (100 µM) dissolved in the bath solution and by mathematical subtraction of the residual current from the control.

Outside-out experiments were performed to isolate the single tmCLIC1 channel using two independent protocols. First, the channel was identified using a voltage-step protocol from − 40 mV to + 40 mV (20 mV steps). Next, the membrane was clamped at 0 mV, and blockers (metformin followed by IAA94) were perfused after at least 3 min of recording.

In time-course experiments, the holding potential was set at −50 mV and, every 5 s, a + 100 mV voltage step was applied. The current was measured at the end of the 800 ms voltage step. Once the current amplitude reached a constant value, metformin and/or IAA94 were perfused, and the bath solution was washed when needed. Analyses were performed using Clampfit 10.2 (Molecular Devices) and OriginPro 9.1. The solutions used were:

Bath solution: 140 mM NaCl, 5 mM KCl, 10 mM Hepes, 5 mM glucose, 2 mM CaCl_2_, 1 mM MgCl_2_, pH 7.4.

Pipette solution (whole cell): 135 mM KCl, 5 mM NaCl, 10 mM Hepes, 1 mM MgCl_2_, 2 mM CaCl_2_, pH 7.4. (outside-out): 120 mM K gluconate, 20 mM KCl, 5mM TEACl, 10 mM Hepes, 0.1 mM CaCl_2_, pH 7.

### Cyclin E1 expression

Cells were synchronized with PD0332991 (24 h) and released ± metformin (5 mM) to evaluate Cyclin E1 expression.

### Recombinant protein purification

Rosetta (DE3) *E. coli* cells were transformed with pET28a-CLIC1 WT or R29A plasmid, grown in continuous shaking (270 rpm) and, once the bacterial culture reached an OD = 1, protein expression was induced with 1mM IPTG for 3 h. Cells were then collected and lysed by sonication, and the soluble fraction was isolated through centrifugation (30 min, 4 °C, 16,000 x g).

This fraction was passed through a HisTrap Nickel column to isolate the protein carrying His-tag sequence. This eluate was used to perform a second chromatographic run in a Superdex 75 column to clean the recombinant protein and solve it in its final buffer (150 mM NaCl, 10 mM HEPES, pH 7.4). Protein concentration was between 1 and 5 mg/ml.NMR Binding Studies.

All spectra were acquired at 25 °C using a Bruker 600 MHz spectrometer. 50 µM metformin was analyzed alone and with 10 µM recombinant CLIC1 (WT or R29A) in PBS (pH 7.4, 10% D2O). WaterLOGSY, T2-filter, and 1D 1 H experiments were conducted.

### In-Cell NMR

Clic1^−/−^ and rescued cells were prepared at 6.5 × 10^6^ cells/mL in D2O-buffered PBS with 2 mM metformin. WaterLOGSY and T2-filter experiments were conducted at 37 °C using a CryoProbe.

### Zebrafish orthotopic xenografts

At 48 hpf, embryos were anesthetized and injected with ~ 150–200 fluorescent tumor cells. Embryos were treated ± 5 mM metformin. Tumor growth was quantified using ImageJ at the time of injection and 3 days post-injection. Zebrafish experiments were conducted up to 5 days post-fertilization (dpf), before independent feeding. They therefore did not require approval by the institutional or national animal care authorities, in accordance with Directive 2010/63/EU and applicable local regulations.

### Mouse orthotopic GBM model

GL261 WT or R29A cells (10^5^) were stereotaxically injected into the caudate nucleus of C57BL/6 N female mice. Metformin (10 mM) was supplied via drinking water from 1 week prior to injection until the endpoint. Brains were harvested and flash-frozen for analysis. All animal procedures were performed under contract by a certified external laboratory (Charles River Laboratories, Germany, project code P1146A). Animals have been randomized in several experimental groups: 5 mice injected with GL261 wild type, 5 mice injected with GL261 wild type treated with metformin, 5 mice injected with GL261 R29A mutated, 5 mice injected with GL261 R29A mutated treated with metformin.

### Metformin brain concentration

 Gas Chromatography-Mass Spectrometry (GC-MS) was used to measure brain metformin levels, using derivatized standards and a deuterated metformin standard as an internal control, as previously described [32]. Briefly, samples were lyophilized, extracted in methanol, and purified via solid-phase extraction. A 25 m × 0.2 mm × 0.3 μm cross-linked methyl silicone gum column (Agilent Technologies, US) was used. The analysis was performed using a thermal gradient, with an initial oven temperature of 120 °C, increased to 160 °C at 10 °C/min, then ramped to 175 °C at 15 °C/min, and finally to 220 °C at 20 °C/min. The analyses were performed in selected-ion monitoring (SIM) mode using a cool-on-column injector at constant flow. The m/z = 303 (derivatized metformin hydrochloride) and m/z = 309 (derivatized deuterated derivatized metformin hydrochloride) ions were monitored. The injected volume was about 1 µL, and the samples were stored at − 20 °C until injection time.

### In silico docking of metformin to CLIC1

The docking pipeline was performed using the Schrödinger Maestro release 2023-3 (Maestro, Schrödinger, LLC, New York, NY, 2023). The structure of human CLIC1 in its soluble dimeric form was obtained from the Protein Data Bank (code 1RK4) [33]. The structure was prepared by adding missing atoms and assigning the most likely protonation state for the ionizable group (pH 7) using the Protein Preparation Wizard tool [34]. The structure was then subjected to energy minimization using the OPLS force field [35] to resolve steric clashes and unfavourable interactions. The metformin molecule was rebuilt from the SMILES (PubChem CID 4091), ligand geometries were optimized using the LigPrep tool [34] and protonation states at pH 7 were generated using Epik [36]. The docking cubic grid was designed on protein chain A, with an edge of 22 Å, centered at coordinates (42.193, 9.568, 34.707). The docking was performed using GlideXP [37], allowing flexible ligand sampling. The seven distinct top-ranked poses of metformin were analyzed for their molecular interactions with the target protein. Protein-ligand interaction diagrams were generated using the Ligand Interaction tool from the Schrödinger Maestro suite, with a distance cutoff of 3 Å from the ligand atoms. Images were created using the open-source version of PyMOL (Schrödinger, LLC). The prepared system and the best poses of metformin are available on Zenodo (DOI: 10.5281/zenodo.14710698)(38).

### Statistical analysis

All data were plotted using GraphPad Prism 7 software (GraphPad Software Inc., San Diego, California). All mean values, standard deviation, and standard errors were calculated, and statistical analysis was performed with the same software. An unpaired t-test analysis was used to compare data between two different conditions. A one-way ANOVA was used to compare more than two groups within the same experimental condition. When the experimental conditions were more than one, a two-way ANOVA test was performed. At least 3 independent replicates were used for each condition in all experiments. *p* < 0.05 was considered statistically significant.

## Results

### Metformin affects gscs’ proliferation and viability via tmCLIC1

**Fig. 1:**
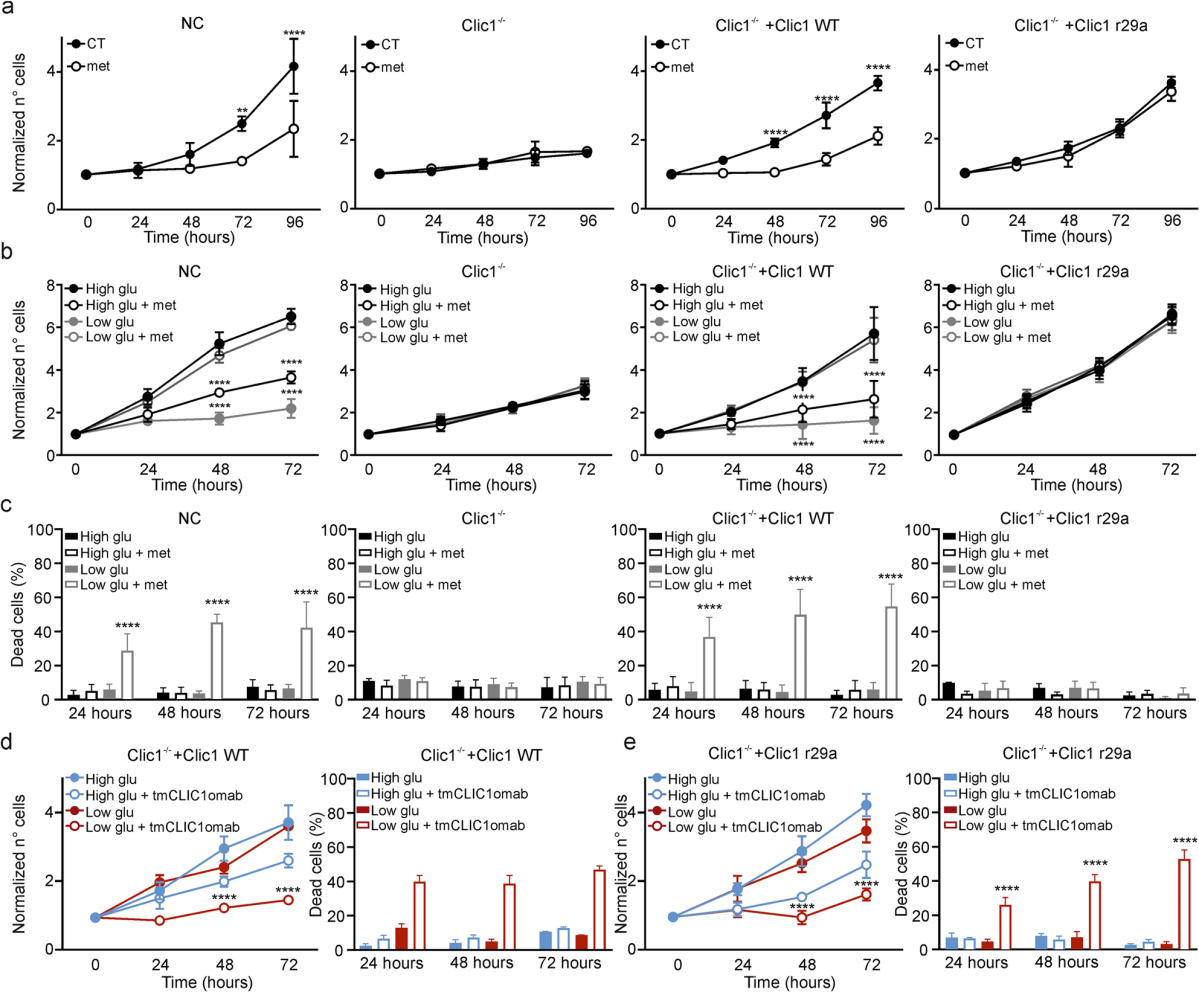
Metformin exerts its effects only when wild-type CLIC1 is expressed. (**a**): GBM1 growth curves over 96 hours in the absence (black circles) or presence (empty circles) of 5 mM metformin in NC, Clic1^-/-^, Clic1^-/-^+Clic1 WT, and Clic1^-/-^+Clic1 R29A. (NC: CT, 24–96 hours, *n*=4; Met, 24–96 hours, *n*=7. 72 hours, **p=0.0011; 96 hours, ****p<0.0001. Clic1^-/-^ CT, 24–72 hours, *n*=4; 96 hours, *n*=2; Met, 24–72 hours, *n*=7; 96 hours, *n*=3. Clic1^-/-^+Clic1 WT: CT, 24, 48, 96 hours, *n*=8; 72 hours, *n*=7; Met, 24 hours, *n*=5; 48–96 hours, *n*=6; 48–96 hours, ****p<0.0001. Clic1^-/-^+Clic1 R29A: CT, 24 hours, 96 hours, n=8; 48 hours, 72 hours, *n*=5; Met, 24 hours, *n*=5; 48–96 hours, *n*=6. Means ± SD, two-way ANOVA, Sidak’s test.) (**b**): GBM1 growth curves over 72 hours in high (black) and low (gray) glucose, with or without 5 mM metformin, in NC, Clic1^-/-^, Clic1^-/-^+Clic1 WT, and Clic1^-/-^+Clic1 R29A. (NC, low Glu vs high Glu: *n*=4; 48–72 hours, ****p<0.0001. Clic1^-/-^: *n*=4. Clic1^-/-^ + Clic1 WT: *n*=7, 48–72 hours, ****p<0.0001. Clic1^-/-^+ Clic1 R29A: *n*=6. Means ± SD, two-way ANOVA Tukey’s test.) (**c**): Dead cells percentage shown (**a**). (NC: n=4, 24–72 hours, ****p<0.0001. Clic1^-/-^+Clic1 WT: *n*=7, 24–72 hours, ****p<0.0001. Mean ± SD, two-way ANOVA Tukey’s test.) (**d**): (left) GBM3 growth curves over 72 hours in high (blue) and low (dark red) glucose, with or without 3.5 µg/ml tmCLIC1omab, in Clic1^-/-^+Clic1 WT (*n*=3, 24 hours, **p=0.0043; 48 hours, **p=0.0021; 72 hours, ****p<0.0001; mean ± SD, two-way ANOVA Tukey’s test.) (right) Dead cells percentage shown in the left panel (*n*=3; 24–72 hours,****p<0.0001; mean ± SD, one-way ANOVA Tukey’s test.) (**e**): (left) GBM3 growth curves with Clic1^-/-^+Clic1 R29A in the same conditions as (**d**) (*n*=3, 48–72 hours, ****p<0.0001; mean ± SD, two-way ANOVA Tukey’s test.) (right) Percentage of dead cells shown in the left panel (*n*=3; 24–72 hours,****p<0.0001; mean ± SD, one-way ANOVA Tukey’s test.)

To evaluate the interaction between metformin and tmCLIC1, three patient-derived GSC-enriched cultures (GBM1-3) and one murine glioma cell line (GL261) lacking CLIC1 expression (Clic1^−/−^) were generated using CRISPR-Cas9 technology. After confirming the absence of endogenous CLIC1, the clones were rescued with two different forms of CLIC1: wild type (WT) and the arginine-to-alanine 29 mutant (R29A), which, based on crystallography studies, is hypothesized to affect the potential metformin binding site of tmCLIC1 [29]. In the three GBM cell lines and murine GBM GL261 cells, we assessed CLIC1 expression levels, tmCLIC1 translocation, and measured its ion conductance in CRISPR-Cas9 negative control (NC), Clic1^−/−^, and rescued cells (Supplementary Fig. 1), confirming the efficacy of gene deletion and rescue. We then evaluated the proliferation rates of these cell populations in 2D and 3D models over 96 h, with or without 5 mM metformin (Fig. 1 and Supplementary Fig. 2). The drug significantly hindered the proliferation of NC GBM1 cells (Fig. 1a). Clic1^−/−^ untreated cells showed a reduced proliferation rate comparable to that of metformin-treated NC cells, but metformin treatment was ineffective on knockout cells. The rescued expression of WT or R29A CLIC1 protein fully restored cellular proliferation. Notably, WT rescued cells regained sensitivity to metformin similar to NC cells (metformin-treated NC vs. metformin-treated Clic1^−/−^ + CLIC1 WT, n.s. p value = 0.5683), while R29A rescued cells’ growth was unaffected by the drug (metformin-treated NC vs. metformin-treated Clic1^−/−^ + CLIC1 R29A, *p-value = 0.0164). The same effect was seen in 3D cultures (Supplementary Fig. 2a). Additionally, we tested the impact of tmCLIC1omab^®^, a monoclonal antibody targeting the transmembrane form of CLIC1, on GBM proliferation to demonstrate further that tmCLIC1 function is crucial for GSC growth. tmCLIC1omab showed the same phenotype in both WT and R29A CLIC1-rescued cells (Supplementary Figs. 2b and c), indicating that the antibody and metformin act on distinct sites of tmCLIC1. We previously reported that metformin cooperates with low glucose to induce cell death in several solid tumors via activation of the CIP2A-GSK3β-MCL1 axis, with metformin treatment leading to CIP2A downregulation [38]. Therefore, we tested whether GBM1-3 are sensitive to the combination of metformin and low glucose (2.5 mM) by measuring proliferation and cell death rates. As seen in other solid tumors, glioblastoma NC cells exhibited a glucose-dependent switch from a cytostatic to a cytotoxic effect of metformin. Moreover, both Clic1^−/−^ and R29A-rescued cell lines were fully resistant to metformin in the presence of low glucose, a cytotoxic combination, indicating that tmCLIC1 is essential for mediating metformin’s cytotoxic effect (Fig. 1b and c). This phenotype was also observed in GBM2, GBM3, and GL261 cells (Supplementary Figs. 2 g-i). Furthermore, tmCLIC1omab^®^ treatment mimicked the effect of metformin combined with low glucose in both WT and R29A rescued cell lines (Figs. 1 d and e).

**Fig. 2:**
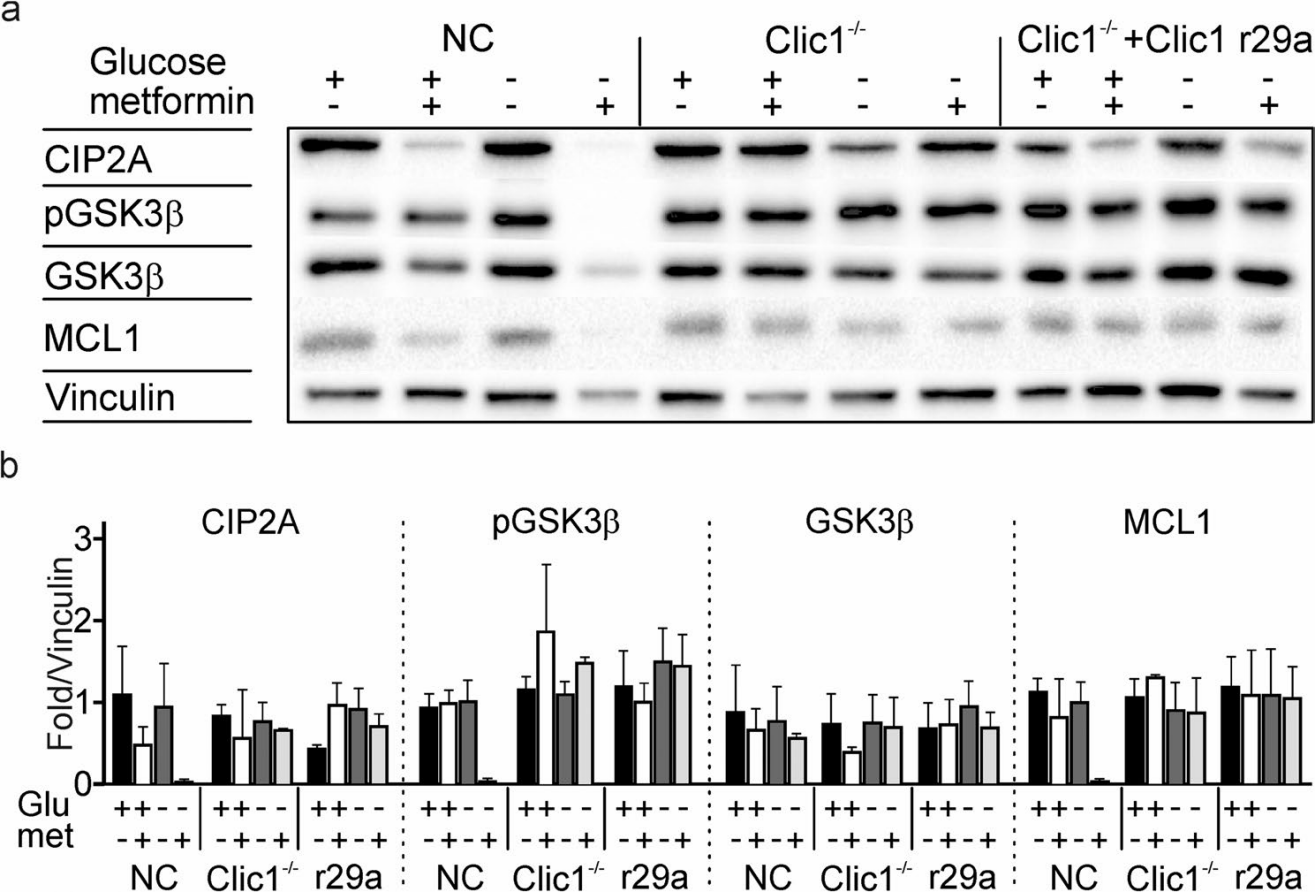
tmCLIC1 inhibition under hypoglycemia affects CIP2A-GSK3b-MCL1 axis. (**a**): Representative Western blot of the expression of the CIP2A-GSK3b-MCL1 axis in NC, Clic1^-/-^, and Clic1^-/-^+Clic1 R29A samples after 72 hours of culture with or without metformin in high or low glucose conditions. (**b**): Quantification of Western blots for CIP2A, phosphorylated GSK3b (pGSK3b), GSK3b, and MCL-1 from cells cultured in high (black filled column) and low (dark gray filled column) glucose. Metformin 5 mM was added in both high glucose (white column) and low glucose (light gray column). (NC: CIP2A high glucose vs. low glucose met **p=0.0023; low glucose vs. low glucose met **p=0.0083. pGSK3b: high glucose vs. low glucose met * *p*=0.0193; high glucose met vs. low glucose met * *p*=0.0124; low glucose vs. low glucose met * *p*=0.0103. MCL-1: high glucose vs. low glucose met ** *p*=0.0053; low glucose vs. low glucose met * *p*=0.015. Mean ± SD, two-way ANOVA, Tukey’s multiple comparison test. *n* = 3

We then examined whether, in GBM1 cells, the combination of metformin and low glucose activated the CIP2A-GSK3β-MCL1 signaling pathway, leading to cell death [38]. As expected, metformin with low glucose levels caused dephosphorylation of GSK3β and subsequent degradation of MCL1 through reduced CIP2A, consistent with increased cell death (Fig. 2). Remarkably, in Clic1^−/−^ and R29A-rescued cell lines, metformin did not decrease CIP2A or downstream phosphorylation of GSK3β (Ser9) and MCL1, which remained unaffected (Fig. 2a). Conversely, R29A-rescued cells show an impaired CIP2A-GSK3β-MCL1 axis in the presence of tmCLIC1omab^®^, emphasizing a key role for tmCLIC1 in this pathway (Supplementary Fig. 2 l). As a control for the specificity of the R29A mutation’s effect, we rescued Clic1^−/−^ cells with a point mutation in Lys37 (K37A), the only other positively charged amino acid in the tmCLIC1 transmembrane region besides Arg29. Despite this mutation, cells remain sensitive to metformin treatment (Supplementary Fig. 2d-f). This highlights the importance of Arg29 as the specific residue needed for metformin binding and activity.

### Metformin causes GSCs to accumulate in the G1 phase

We previously reported that tmCLIC1’s activity depends on the timing of signals that control its insertion and removal from the membrane, and its activity is essential during the G1/S cell cycle transition [28]. Therefore, we examined the effects of tmCLIC1 and metformin on the timing of the G1/S transition across four genetic backgrounds of GBM1 cells. Cyclin E1 accumulates at the G1/S phase boundary and is significantly degraded as cells pass through the S phase. We measured Cyclin E1 levels in early G1 synchronized cells at various time points to track cell cycle progression. The dynamics of Cyclin E1 followed the same pattern in all clones, except for Clic1^−/−^ cells (Supplementary Fig. 3a), which exhibited delayed cell cycle progression, consistent with their slower proliferation (Fig. 1). As expected, metformin-treated NC and Clic1 WT-rescued cells exhibited a delayed peak of Cyclin E1. Clic1 R29A-rescued cells did not alter the timing of Cyclin E1 levels after metformin treatment, confirming their insensitivity to the drug (Supplementary Fig. 3a). Additionally, tmCLIC1omab^®^ mimicked metformin’s effects in NC cells and was also effective in R29A-rescued cells (Supplementary Fig. 3b). Furthermore, GBM2 and GBM3 cells showed a phenotype similar to GBM1 when treated with metformin (Supplementary Fig. 3c), suggesting that the pathway is conserved across multiple cell cultures.

**Fig. 3:**
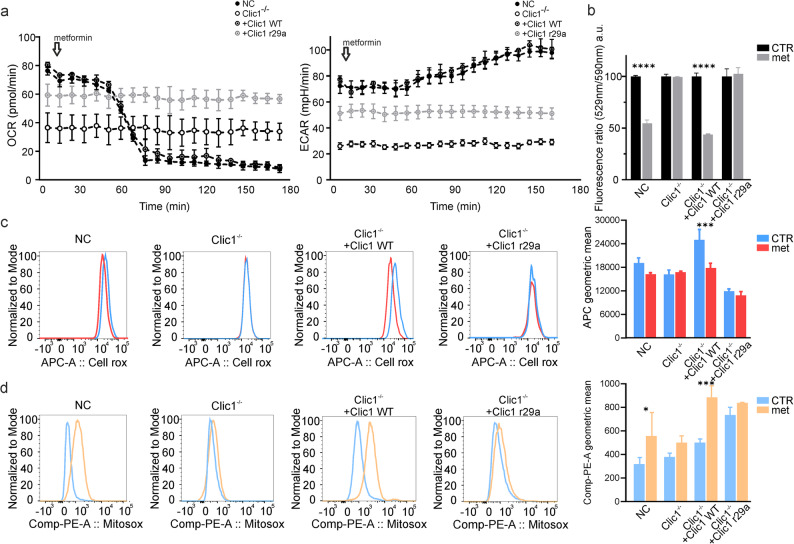
Metformin’s effect on metabolism depends on CLIC1. (**a**): Oxygen consumption rate (left) and extracellular acidification rate (right) in GBM1, used as indicators of oxidative phosphorylation and glycolytic activity, respectively, after an acute injection of 10 mM metformin. The samples include NC (black circles), Clic1^-/-^ (empty circles), Clic1^-/-^ + Clic1 WT (black circle dots), and Clic1^-/-^ + Clic1 R29A cells (grey circle dots). Each data point was recorded every 6 minutes. (**b**): Mitochondrial membrane potential measured in GBM1 using a JC-1 probe across NC, Clic1^-/-^, Clic1^-/-^ + Clic1 WT, and Clic1^-/-^ + Clic1 R29A, in the absence (black columns) or presence (grey columns) of 5 mM metformin. *N*=2;****p<0.0001; data expressed as mean ± SD, analyzed with two-way ANOVA and Sidak’s multiple comparison test. (**c**): (left) Representative FACS plot of CellROX^TM^ Deep Red Reagent fluorescence for detecting oxidative stress in GBM3. Cells were incubated for 3 hours without (blue) or with (red) 5 mM metformin. (right) Average fluorescence intensity of CellROX^TM^ Deep Red Reagent. *N*=3, Clic1^-/-^ + Clic1 WT: CT vs Met; ***p=0.0002; data shown as mean ± SD, analyzed using two-way ANOVA and Tukey’s multiple comparison test. (**d**): (left) Representative FACS analysis of MitoSOX^TM^ Red Indicator fluorescence for mitochondrial superoxide detection in GBM3. Cells were incubated for 3 hours without (blue) or with (yellow) 5 mM metformin. (right) Mean fluorescence intensity of MitoSOX^TM^ Red Indicator. *N*=4, NC: CT vs Met, *p=0.0361. Clic1^-/-^ + Clic1 WT: CT vs Met, ***p=0.0005. Data expressed as mean ± SD, analyzed with two-way ANOVA and Tukey’s multiple comparison test

### Metabolism modulation by metformin depends on CLIC1

Metformin is recognized as an inhibitor of OXPHOS in many cancer cells. Therefore, we examined how GBM cell metabolism (including mitochondrial respiration and glycolysis) changed after acute treatment with a high concentration of metformin (10 mM) in NC, Clic1^−/−^, rescued WT, and rescued R29A cells. Both NC and rescued WT cells exhibited similar mitochondrial respiratory rates; following metformin injection, they experienced a rapid decrease in oxygen consumption and a gradual increase in extracellular acidification, consistent with the presumed inhibition of OXPHOS. Conversely, Clic1^−/−^ cells showed a significant decline in basal metabolism and appeared unaffected by metformin treatment. Rescued R29A cells had a basal metabolism similar to rescued WT but did not respond to metformin (Fig. 3a, for GBM1; Supplementary Figs. 4a and c, for GBM2 and GBM3). Cells treated with tmCLIC1omab^®^ showed a comparable effect to metformin (Supplementary Figs. 4e-g, GBM2, and GBM3), indicating that disrupting tmCLIC1 function quickly affects mitochondrial respiration.

To further demonstrate that the metformin-tmCLIC1 interaction triggers a stress cascade involving tmCLIC1, mitochondrial activity, and proliferation in GBM, we measured mitochondrial membrane potential using a JC1 probe 30 min after metformin treatment. NC and rescued WT quickly responded to metformin, showing a decrease in mitochondrial potential, while Clic1^−/−^ and R29A were unaffected by the treatment (GBM1: Fig. 3b; GBM2 and GBM3: Supplementary Fig. 4b and d).

To evaluate whether metformin influences oxidative stress, we measured intracellular reactive oxygen species (ROS) and mitochondrial superoxide (O^2−−^) production in four genetic backgrounds of GBM1-3 cells, with or without metformin (Fig. 3c-d and Supplementary Fig. 4h-k). Acute metformin treatment reduced ROS production in NC and WT-rescued populations but did not affect Clic1^−/−^ and R29A-rescued cells (Fig. 3c). Similarly, metformin did not alter O^2−^ generation in Clic1^−/−^ and R29A-rescued clones. However, in the presence of tmCLIC1 protein, there was a notable increase in superoxide production, likely indicating acute mitochondrial stress (Fig. 3 d). Additionally, GBM1 Clic1^−/−^ and R29A-rescued cells treated with IACS and niclosamide, both mitochondrial complex I inhibitors, exhibited strong cytotoxicity (Supplementary Figs. 2j-k). These results support the conclusion that the upstream effect of metformin on OXPHOS is mediated by tmCLIC1 at the plasma membrane.

### Evidence of direct binding between metformin and CLIC1 protein

**Fig. 4:**
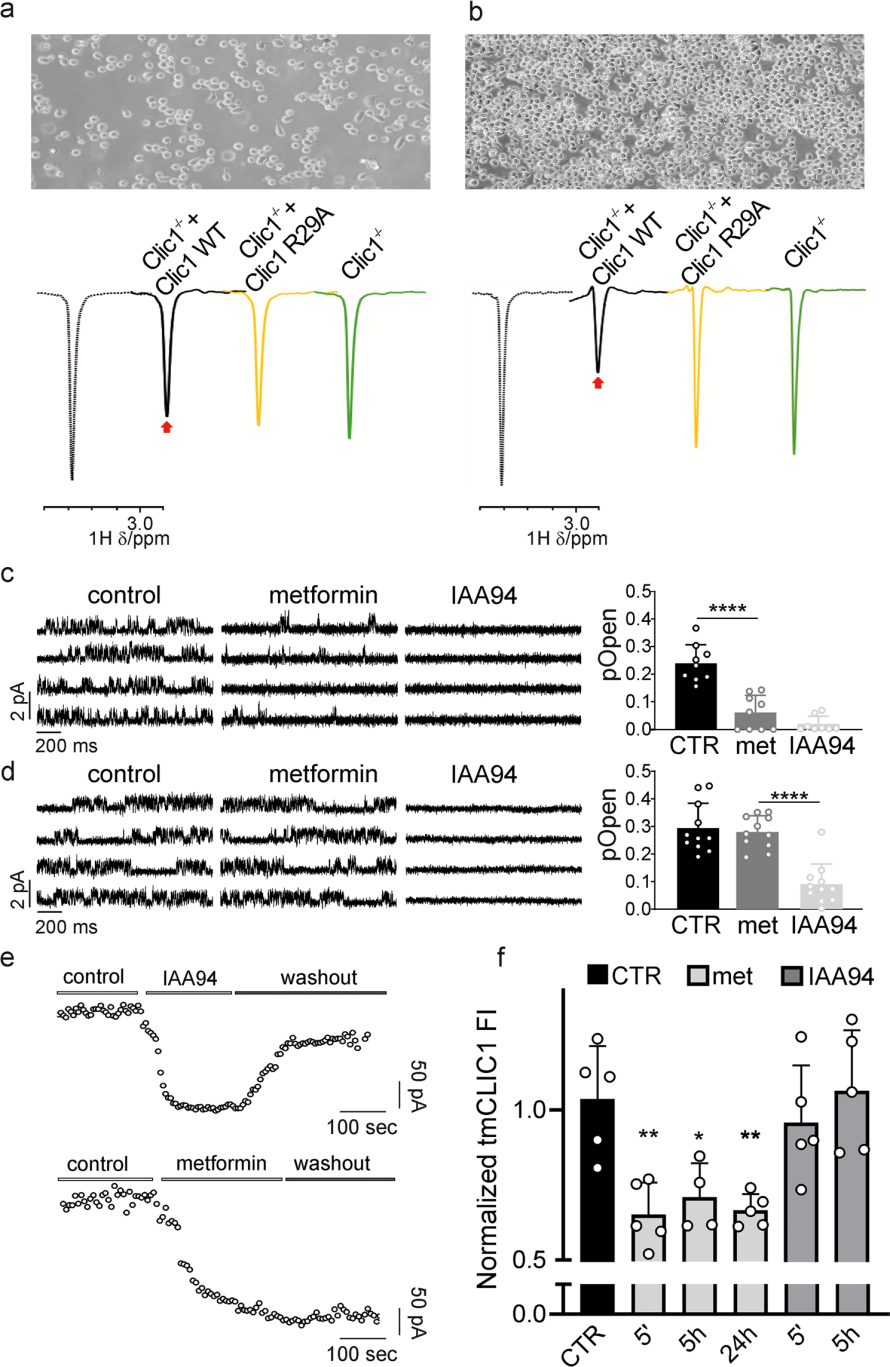
Metformin directly interacts with tmCLIC1. (**a**): WaterLOGSY NMR spectra of 2 mM metformin in the absence (dashed line) or presence of Clic1^-/-^ (green), Clic1^-/-^ + Clic1 WT (black), and Clic1^-/-^ + Clic1 R29A (yellow) cells. Arrows highlight signal changes, showing a significant effect of metformin binding in the presence of Clic1^-/-^+ Clic1 WT cells compared with Clic1^-/-^ + Clic1 R29A and Clic1^-/-^ cells. (**b**): same experiment as in (**a**) performed with twice the number of cells. (**c**): Representative traces of GBM3 Clic1^-/-^ + Clic1 WT outside-out experiments during perfusion of vehicle (CTR) or indicated compounds (left). Quantification of tmCLIC1 single-channel open probability (right). CTR and met *n*=9; IAA94, *n*=8;****p<0.0001; mean ± SD, one-way ANOVA, Tukey’s multiple comparison test. (**d**): Same experiment as (**c**) in GB3 Clic1^-/-^ + Clic1 cells (left). Quantification of tmCLIC1 single-channel open probability (right). *n*=11;****p<0.0001; mean ± SD, one-way ANOVA, Tukey’s multiple comparison test. (**e**) Representative time-course of whole-cell currents in WT cells. Cells were stimulated every 5 seconds with an 800 ms, +60 mV test potential from the resting potential. Once the current amplitude reached a constant value, 100 mM IAA94 (top) and 10 mM metformin (bottom) were perfused, then washout was performed in both experimental conditions. (7) Relative fluorescence intensity of tmCLIC1 in WT cells after several times of metformin (grey column) and IAA94 (dark grey column) incubation. Mean± SD, one-way ANOVA, Tukey’s multiple comparison test (CTR *n*=5, met 5’ = 5, met 5 h *n*=4, met 24 h *n*=5, IAA94 5’ *n*=5, IAA94 5 h *n* =5. CTR vs met 5’ **, *p*=0.0060, CTR vs met 5 h * *p*=0.0378; CTR vs met 24 h ** *p*=0.0086; CTR vs IAA94 5’ n.s. *p*= 0.9614; CTR vs IAA94 5 h n.s. *p*=0.9997)

To confirm metformin’s direct binding to tmCLIC1, WaterLOGSY (Water-Ligand Observation with Gradient Spectroscopy) [39, 40] and ^1^H T_2_ filter (transverse relaxation filter) [41] NMR experiments were performed on metformin (2 mM) in the absence or presence of Clic1^−/−^, Clic1^−/−^ + Clic1WT, or Clic1^−/−^ + Clic1R29A cells (Fig. 4a, Supplementary Fig. 5 d). An apparent binding effect of metformin was observed in the presence of WT-rescued cells, whereas only a modest interaction was detected with Clic1^−/−^ cells. The residual binding observed with Clic1^−/−^ cells may reflect binding to secondary metformin targets; however, the markedly-reduced binding observed with R29A-rescued cells suggests that this specific mutation significantly impairs metformin’s ability to bind CLIC1. These findings were further validated by increasing the number of cells analyzed in the NMR experiments, which more clearly highlighted metformin binding in WT-rescued cells compared with the background interaction observed in Clic1^−/−^ cells (Fig. 4b, Supplementary Fig. 5 d). Additional NMR experiments were performed on isolated recombinant WT and R29A-mutated proteins (see Materials and Methods) to confirm the direct molecular interaction between metformin and CLIC1. WaterLOGSY and ^1^H T_2_ filter NMR data showed that metformin binds to the WT form of CLIC1, but not to the R29A mutant (25 °C, Supplementary Fig. 5e). These results provide molecular evidence that replacing a charged, hydrophilic residue (R) at position 29 with a hydrophobic one (A) severely compromises metformin’s ability to bind CLIC1.

To provide evidence of a direct effect of metformin on tmCLIC1 activity, we performed patch clamp experiments to monitor how tmCLIC1 activity changes over time with metformin perfusion. The tmCLIC1-specific inhibitor IAA94 served as a control. Metformin treatment decreased whole-cell currents in NC and WT-rescued cells (Supplementary Fig. 5a). Consistent with our previous results, in R29A-rescued cells, metformin perfusion did not reduce the tmCLIC1-associated current (Supplementary Fig. 5a).

To gain a clearer understanding at the molecular level, we performed single-channel outside-out experiments under the same conditions (see Materials and Methods). In WT-rescued cells, the tmCLIC1 single-channel current was blocked by metformin and was not further affected by IAA94 (Fig. 4c; Supplementary Fig. 5). Conversely, metformin did not influence the single-channel activity of R29A-rescued cells, which IAA94 completely inhibited. These findings suggest that metformin directly binds to tmCLIC1 in GSCs (Fig. 4 d; Supplementary Fig. 5). We then tested whether the phenotype observed after adding a single inhibitor persisted after removing both IAA94 and metformin. Whole-cell time-course experiments on WT cells involved perfusing with either IAA94 or metformin, followed by a single wash once the current stabilized. As expected, both inhibitors significantly reduced the whole-cell current. However, while washing out, IAA94 restored over 60% of the initial current, the effect of metformin was irreversible (Fig. 4e). Structural studies [42] indicate that IAA94 affects cysteine 24, which faces the external membrane, whereas the metformin binding site is located in the proposed transmembrane segment of the protein. The irreversible effect of metformin on the current might relate to a decrease in tmCLIC1 expression on the plasma membrane of GSCs, due to its direct binding with the protein. To verify this, tmCLIC1 fluorescence was measured on the plasma membrane of cells incubated with metformin or IAA94 for various durations. After each treatment, the inhibitors were removed, and tmCLIC1 fluorescence was assessed 72 h later using the Fluorescence Intensity assay. Cells treated with metformin showed significantly lower tmCLIC1 levels compared to untreated cells after just 5 min of incubation, while IAA94-treated cells maintained tmCLIC1 levels similar to controls at all time points (Fig. 4f).

### Metformin affects tumor expansion in zebrafish embryos and mouse glioblastoma models

**Fig. 5:**
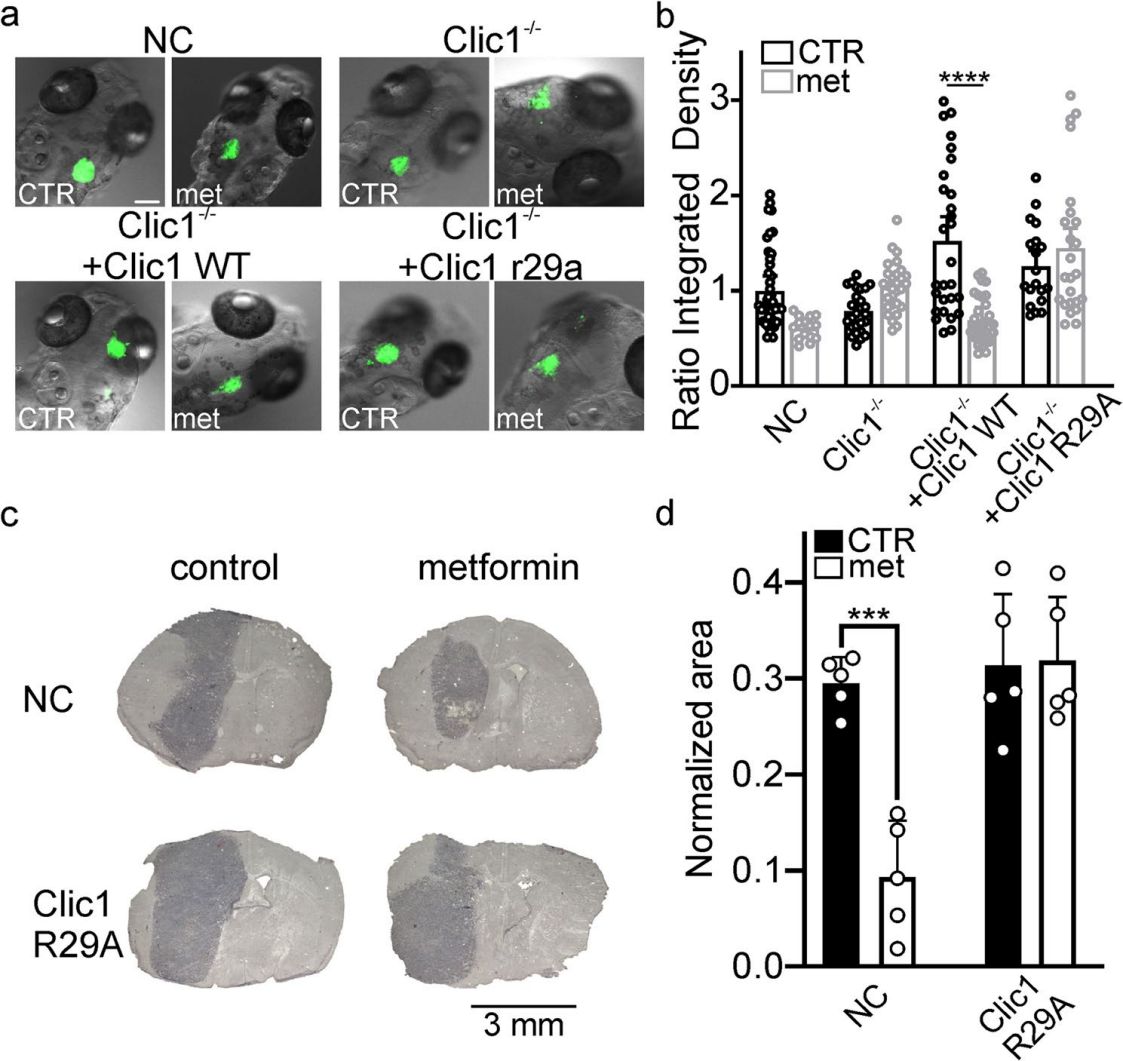
Metformin impairs GBM xenografts' growth in zebrafish and in immunocompetent mice. (**a**): Representative images of the expansion of the tumor mass at 72 h post-injection in the zebrafish embryonic brain injected with NC, Clic1^-/-^, Clic1^-/-^ + Clic1 WT, Clic1-/- +Clic1 R29A, or GBM1 cells in absence or presence of 5 mM metformin dissolved in embryo medium. Scale bar 100 µm. (**b**): Quantification of green fluorescence integrated density from the tumor mass in absence (black) or presence (grey) of metformin. Each dot on the graph represents the expansion of the tumor mass measured in a single embryo's brain at four hpi and three dpi. NC: CTR, *n*=44, Met, *n*=17; n.s. p=0.1036. Clic1^-/-^ + Clic1 WT: CTR, *n*=28, Met, *n*=31; ****p<0.0001. Mean ± SD, two-way ANOVA, Tukey's multiple comparison test. (**c**): Representative hematoxylin-stained histological images from mice injected intracranially with GL261 NC (top) and GL261R29A knock-in (bottom) treated (right) or not (left) with 10 mM metformin in drinking water. (**d**): Normalized area of tumoral mass in hematoxylin-stained brain slices of mice treated (white columns) or not (black columns) with 10 mM of metformin (Control, *n*=5; metformin, *n*=5; ***p=0.0003, one-way ANOVA, Tukey's multiple comparison test

To determine whether the effect of metformin persists in in vivo setting, zebrafish embryos were orthotopically injected with GBM1 cells at 48 h post-fertilization. The tumor mass was measured after 72 h in the presence or absence of metformin (5 mM) diluted into the embryos’ water. Consistent with our in vitro observations (Fig. 1), metformin effectively reduced tumor growth in xenografts from NC and WT-rescued cells (Figs. 5a and b) but was ineffective in Clic1^−/−^ cells or with the R29A point mutation (Figs. 5a and b).

To validate these results in an *in vivo* mammalian model, we used GL261 cells, both WT and R29A knock-in cells, to establish an orthotopic and syngeneic murine GBM model. [43]. Metformin was administered in drinking water at 10 mM for up to 50 days, resulting in measurable brain concentrations [32] (average 7.25 nM ± 0.48, n = 12, Table 1). Mice were sacrificed on day 52, and the brains were collected (WT cells n = 6, and Clic1^-/-^ R29A n = 6). Histological analysis showed that tumor invasion in WT-injected brains was significantly reduced in metformin-treated mice compared to untreated mice (Fig. 5c, d). Conversely, Clic1^-/-^ R29A-injected mice showed similar tumor sizes in the brain as WT-injected mice; both treated and untreated conditions were comparable in Clic1^-/-^ R29A-injected brains (Fig. 5c, d).

**Table 1:**
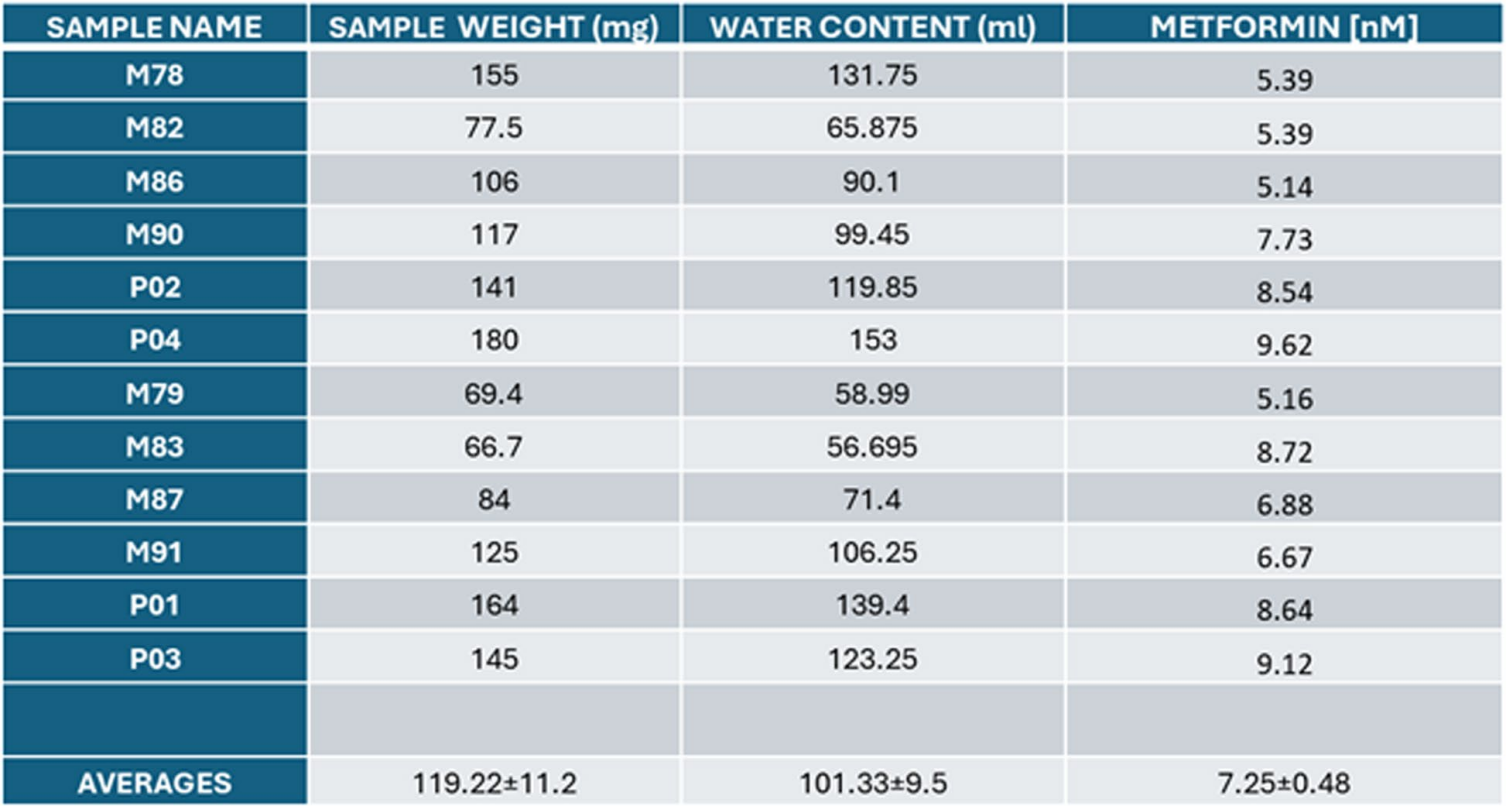
Measurement of metformin concentration in the mouse brain

All the brains were injected with GL261 murine glioblastoma cells. Metformin at 10 mM concentration was supplied to the mice in the drinking water with the new solution every 2 days. Mice were exposed to metformin for 30–50 days (*n* = 12)

### Metformin binds the CLIC1 oxidized dimer in an amino acid pocket coordinated by R29

**Fig. 6:**
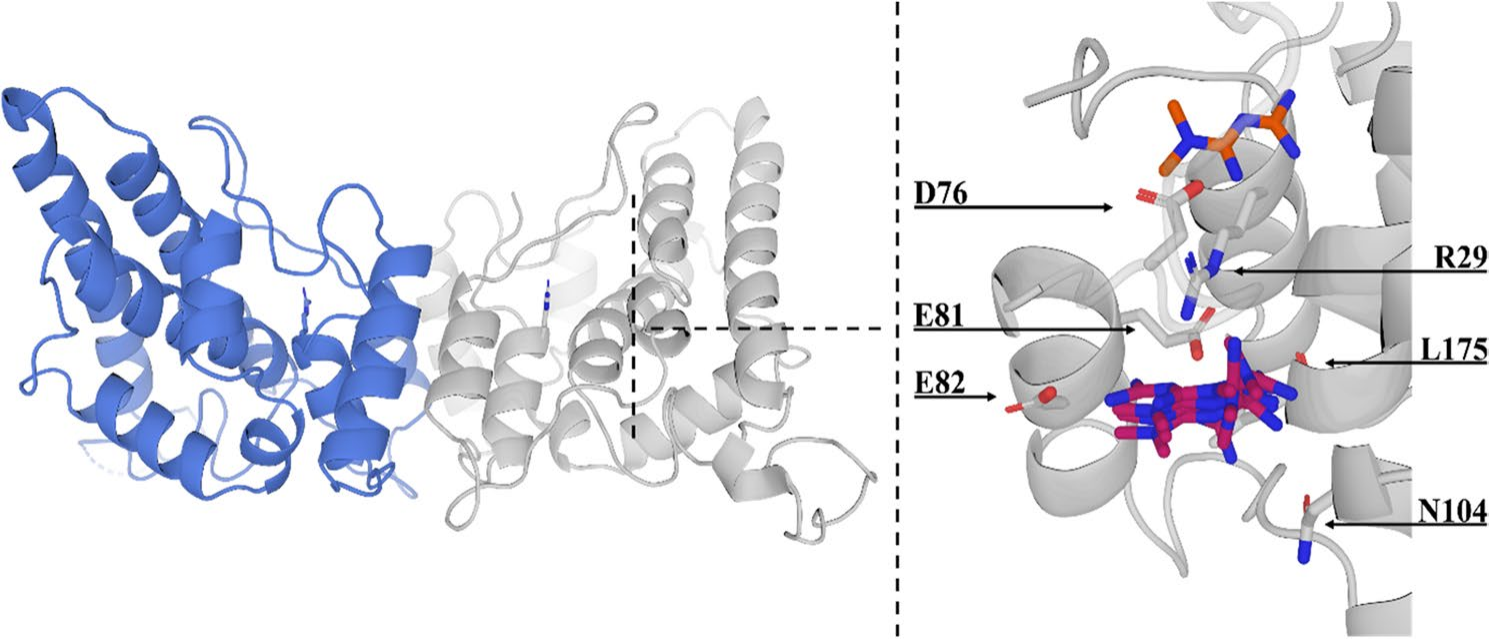
In silico model of CLIC1-metformin interaction. Crystal structure of the human soluble dimeric form of oxidized CLIC1 (left) (PDB ID 1RK4) in cartoon representation, with chain A in light gray and chain B in light blue. Arginine 29 is shown as sticks, with nitrogen (N) atoms and carbon (C) atoms matching their respective chain colors (light gray for chain A and light blue for chain B). Close-up view of the putative metformin binding site on CLIC1 (right), displaying seven docking poses. Metformin poses one to six are superimposed and shown as sticks with C atoms in purple and N atoms in blue, while pose seven, deviating from the main binding region, is shown with C atoms in orange. Relevant residues interacting with metformin across the seven poses (R29, D76, E81, E82, N104 and L175) are displayed as sticks, with C atoms in light gray, oxygen atoms in red, and N atoms in blue. Hydrogen atoms are omitted for clarity

The docking study of metformin with the soluble dimeric form of CLIC1 revealed interactions within the binding pocket, highlighting the potential role of key residues in stabilizing the ligand and maintaining the structural integrity of the binding site (Fig. 6, Supplementary Table 1). Among the seven docking poses analyzed, with scores ranging from − 2.141 to −1.191, metformin was located in the same region of the binding pocket in six out of seven poses, adopting slightly different orientations while maintaining consistent interactions (Fig. 5). In these six poses, E81 was identified as the central residue, forming hydrogen bonds or salt bridges with metformin, suggesting a key role in ligand stabilization. Only pose seven deviated from this pattern, with metformin displaced to another region of the pocket, where it primarily interacted with D76.

The residue R29 directly forms a weak hydrogen bond with the middle nitrogen atom of metformin only in docking pose six; this atom is not protonated at physiological pH and acts as a hydrogen bond acceptor by interacting with hydrogen donors, such as the positively charged guanidinium group of arginine. Besides this possible interaction, R29 mainly contributes to the structural stability of the binding pocket. Its side chain participates in a hydrogen bond network involving D76 through both side chain and backbone interactions, as well as with the side chain of E81, thereby reinforcing the pocket architecture. The hydrophobic environment provided by L175 and A176 supports ligand binding by complementing the polar interactions, with the main chain of L175 forming hydrogen bonds with metformin in four poses. Residue N104 occasionally forms polar interactions with metformin, aiding its stabilization within the binding site. E82 also displayed salt bridge and hydrogen bond interactions with metformin in the two highest-ranking poses (Fig. 6, Supplementary Table 2, Supplementary Fig. 6).

## Discussion

Glioblastoma is one of the deadliest solid tumors, characterized by a high recurrence rate despite surgery and aggressive drug treatments. The focus of new therapies is targeting GSCs, which are believed to drive both tumor growth and relapses. Cancer cells, especially GSCs, have higher energy and biomolecule requirements than healthy cells, enabling them to adapt their metabolism by switching between glycolysis and OXPHOS. As a result, research has focused on strategies to halt tumor energy supply [44, 45], where ion channel dysfunctions play a key role. Our lab identified tmCLIC1 as a potential therapeutic target [27, 28]. The tmCLIC1 protein is constantly overexpressed in the plasma membrane of many solid cancers (47–53), promoting their growth and progression, while it remains largely absent in normal cells. In this work, we demonstrated that blockade of tmCLIC1 in GSCs is not simply involved in the antiproliferative action of metformin [29] but also acts as a privileged receptor for the antidiabetic drug. R29A-mutated tmCLIC1 exhibits biophysical characteristics comparable to the wild-type membrane protein. Although we cannot rule out that metformin has other targets in glioblastoma, in point-mutated (R29A) cancer cells, metformin shows no functional effect. Delivery of metformin to glioblastoma cells bearing the arginine 29 mutation fails to demonstrate its antiproliferative properties.

Metformin impairs mitochondrial respiration, thereby inhibiting tumor cell growth and invasion, only when bound to tmCLIC1. Furthermore, when combined with hypoglycemia, metformin binding to tmCLIC1 disrupts the PP2A-GSK3β-MCL-1 pathway, leading to cancer cell death. However, these effects occur at millimolar concentrations, with electrophysiological measurements showing an affinity of approximately four mM between the drug and tmCLIC1. In vivo studies demonstrated that mice treated with metformin in their drinking water exhibited significantly slower glioblastoma growth, even though brain levels of the drug reached only nanomolar concentrations (Table 1) [46]. This notable discrepancy warrants further investigation. The hypothesis is that tmCLIC1 does not function as a typical ion channel. The structure of tmCLIC1 within the membrane remains unresolved. Several studies suggest that the transmembrane form of the protein is a homocomplex composed of two, three, or even four dimers. Nonetheless, it is unlike any known ion channel, as indicated by its amino acid sequence and the structure of its dimer. The rearrangement of the hydrophilic monomer into a dimer is necessary for the protein to integrate into the membrane [47]. This structure, especially the putative transmembrane segment (aa24-46), does not resemble any known ion channel [36, 48]. Our hypothesis proposes that tmCLIC1 forms a protein complex capable of conducting chloride ions through a transmembrane pathway that is independent of both time and voltage. A flickering kinetic observed during patch-clamp single-channel recordings supports this idea (Fig. 4 C and D). In this model, metformin does not act as a traditional ion channel blocker; instead, it inhibits chloride currents by accumulating within the tmCLIC1 complex through irreversible binding. We have shown that continuous delivery of metformin via drinking water maintains stable drug levels in the brains of mice over time. The antiproliferative effect and sustained brain levels of metformin were not observed when the drug was administered through gavage. We suggest that this accumulation enhances antiproliferative effects at lower concentrations than those required for short-term in vitro treatments. Initial experiments indicate that the concentration of metformin needed to inhibit tmCLIC1 is inversely related to exposure time. We found that tmCLIC1’s interaction with metformin depends on the positively charged residue R29. However, a missing link remains: how can a positively charged molecule like metformin interact with an arginine residue and influence mitochondrial function? Molecular docking simulations revealed that, despite their similar charges, metformin ends up close to R29, highlighting the importance of this amino acid in binding. Nevertheless, this does not explain how the interaction occurs. We examined the environment surrounding R29 and identified a “pocket” of negatively or partially negatively charged amino acids that could facilitate metformin binding (Fig. 6). Inhibitors such as IAA94 and tmCLIC1omab^®^ mimic the effects of metformin, despite binding at different sites. Both agents impair the ion channel function of tmCLIC1, even when R29 is mutated. Disruption of tmCLIC1’s function interferes with the metabolic processes of GSCs. It has been shown that tmCLIC1 is essential for maintaining membrane charge balance, supporting membrane-bound NADPH oxidase activity [28]. We believe that tmCLIC1 plays a vital role in generating reactive oxygen species (ROS), which are crucial for GSC metabolic reprogramming. Moreover, we have demonstrated that tmCLIC1 lies upstream of the PP2A-GSK3β-MCL-1 pathway, thereby ensuring GSC survival. Continuous metformin administration could enhance this cytostatic and cytotoxic process.

## Conclusions

In conclusion, impairing tmCLIC1 with an inorganic molecule, a monoclonal antibody, or metformin weakens glioblastoma cells by significantly reducing energy production. Metformin offers the advantage of being a safe drug with minimal side effects and is already approved by healthcare systems worldwide. However, clinical trials involving metformin have yielded mixed results [47–49]. This study provides insights into the mechanism of interaction between metformin and the tmCLIC1 complex. Our main advancement is the method of delivering metformin. Dissolving the drug in drinking water and maintaining consistent oral intake ensures a steady level of metformin in the brain, enabling the inhibition of tmCLIC1 even at nanomolar concentrations. Impairing tmCLIC1 is essential for reducing tumor aggressiveness, particularly in preventing cancer stem cells from promoting tumor recurrence. This broad suppression of tumor potency allows standard treatments to become more effective and may help limit tumor growth and invasion when used in combination therapy.

## Supplementary Information

Supplementary Material 1.

Supplementary Material 2.

## Data Availability

The datasets used and/or analyzed during the current study are available at: 10.13130/RD_UNIMI/NWY7KY.

## Abbreviations

GBM: Glioblastoma
GSCs: Glioblastoma stem cells
OXPHOS: Oxidative phosphorylation
CLIC1: Chloride Intracellular Channel 1
tmCLIC1: Transmembrane Chloride Intracellular Channel 1
IAA94: Indanyloxyacetic acid 94
Met: Metformin
GBM1: Primary glioblastoma stem cell culture 1
GBM2: Primary glioblastoma stem cell culture 2
GBM3: Primary glioblastoma stem cell culture 3
OCR: Oxygen consumption rate
ECAR: Extracellular Acidification Rate
ROS: Reactive Species of Oxygen
WT: Wild type
R29A: Arginine-to-alanine 29 mutant
NC: Negative Control
Glu: Glucose
CIP2A: Cancerous Inhibitor of Protein Phosphatase 2 A
PP2A: Heterotrimeric protein phosphatase
GSK3β: Glycogen Synthase Kinase 3 Beta
MCL1: Myeloid Cell Leukemia 1
Lys37: Lysin 37
Arg29: Arginine 29
O^2−−^: Mitochondrial superoxide
NMR: Nuclear Magnetic Resonance
E81: Glutamic acid 81
D76: Aspartic acid 76
L175: Leucine 175
A176: Alanine 176
N104: Asparagine 104
E82: Glutamic acid 82
NADPH: Nicotinamide Adenine Dinucleotide Phosphate
