# Correction: Chronic administration of Metformin exerts cytostatic and cytotoxic effects via the PP2A-GSK3β-MCL-1 pathway by inhibiting the tmCLIC1 membrane protein in glioblastoma-initiating cells

**DOI:** 10.1186/s13046-025-03628-9

**Published:** 2026-01-20

**Authors:** Francesca Cianci, Ivan Verduci, Riccardo Cazzoli, Gaetano Cannavale, Guido Rey, Marina Veronesi, Beatrice Balboni, Matteo Ranucci, Luca Maria Giovanni Palloni, Federico Ballabio, Noemi Barsotti, Giorgia Ailuno, Alice Balboni, Sara Baldassari, Gabriele Caviglioli, Carlotta Tacconi, Carlo Camilloni, Stefania Girotto, Alessandro Fantin, Federica Barbieri, Andrea Cavalli, Massimo Pasqualetti, Tullio Florio, Saverio Minucci, Michele Mazzanti

**Affiliations:** 1 Department of Bioscience, University of Milan, Via Celoria 26, Milano, 20133 Italy; 2 IEO, Istituto Europeo di Oncologia, Milano, Italy; 3 Structural Biophysics Facility, Istituto Italiano di Tecnologia, Genoa, Italy; 4 D3 PharmaChemistry, Istituto Italiano di Tecnologia, Genoa, Italy; 5 Computational and Chemical Biology, Istituto Italiano di Tecnologia, Genova, Italy; 6 Department of Internal Medicine, Università di Genova, Genova, Italy; 7 IRCCS Ospedale Policlinico San Martino, Genova, Italy; 8 Department of. Pharmacy, Università di Genova, Genova, Italy; 9 Department of Biology, Unit of Cell and Developmental Biology, University of Pisa, Pisa, 56127 Italy; 10 Present address: 3Brain IT SRL, Genova, Italy; 11 Present address: University of New South Wales, Kensington, Sydney, NSW Australia; 12 Present address: Dept. Pharmacology and Toxicology, University of Zurich, Zurich, Switzerland; 13 Present address: IIT, Genova, Italy; 14 Present address: Vita-Salute San Raffaele University, Milan, Italy; 15 Department of Pathophysiology and Transplantation, University of Milano, Milano, Italy; 16 Department of Experimental Oncology,, IEO, European Institute of Oncology IRCCS, Milan, Italy

**Correction: J Exp Clin Cancer Res 44**,** 312 (2025)**

**https://doi.org/10.1186/s13046-025-03577-3**

Following publication of the original article [1], the authors would like to correct the affiliation of Dr. Saverio Minucci. The details are given below:

**Current affiliation:** ^2^IEO, Istituto Europeo di Oncologia, Milano, Italy

**Correct affiliation:** ^16^Department of Experimental Oncology, IEO, European Institute of Oncology IRCCS, Milan
