# Supplementary material for: Chronic administration of metformin exerts cytostatic and cytotoxic effects via the PP2A-GSK3β-MCL-1 pathway by inhibiting the tmCLIC1 membrane protein in glioblastoma-initiating cells
Source: J Exp Clin Cancer Res. 2025 Nov 24;44:312. doi: 10.1186/s13046-025-03577-3 (PMC12659486; doi:10.1186/s13046-025-03577-3)
Supplement: Supplementary file 1 — Supplementary Material 1. [file 13046_2025_3577_MOESM1_ESM.pdf]

GEL 1

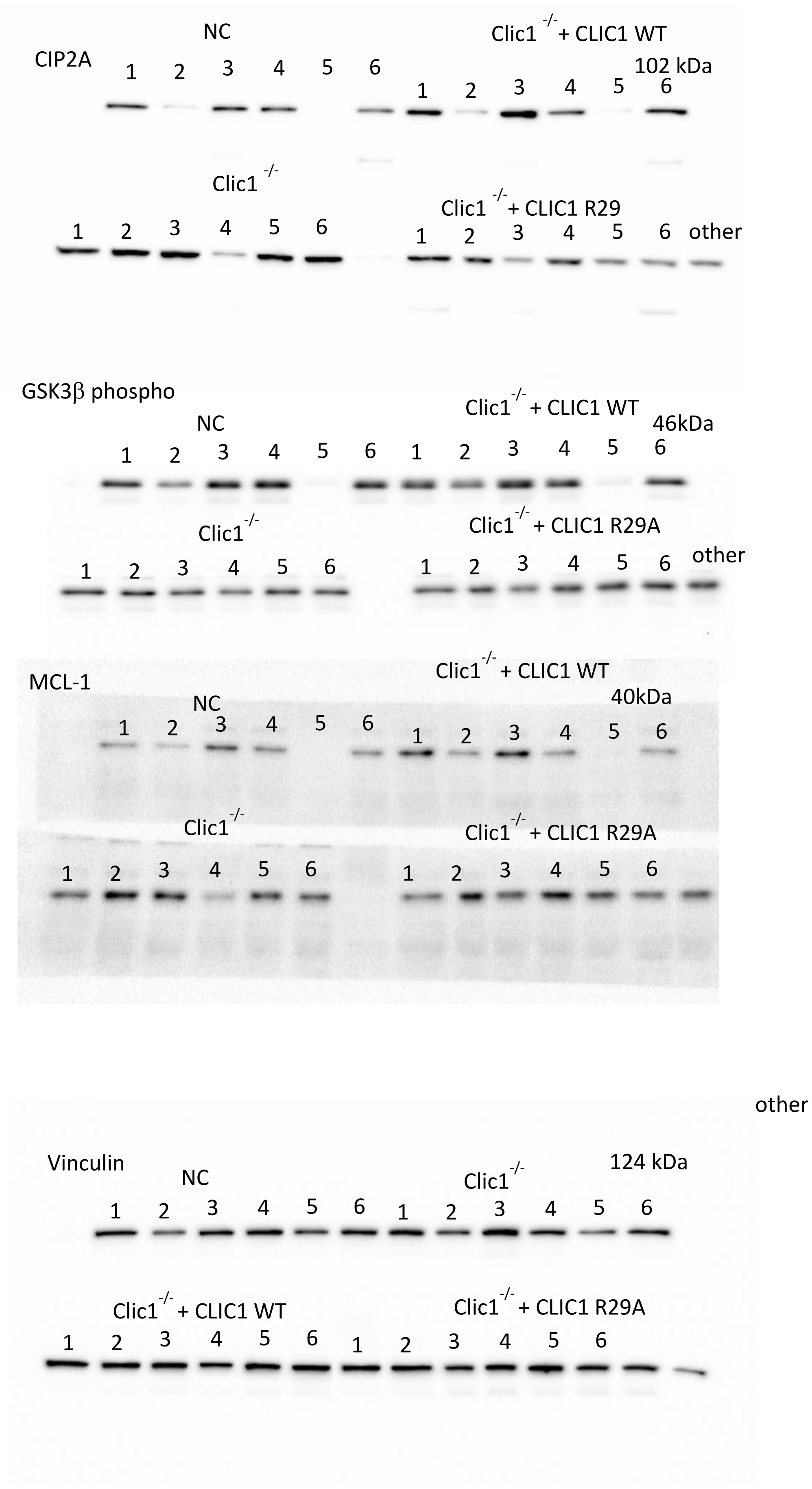
CIP2A
NC
1 2 3 4 5 6
Clic1 -/- + CLIC1 WT
102 kDa
1 2 3 4 5 6
Clic1 -/-
1 2 3 4 5 6
Clic1 -/- + CLIC1 R29
1 2 3 4 5 6 other
GSK3β phospho
NC
1 2 3 4 5 6
Clic1 -/- + CLIC1 WT
46kDa
1 2 3 4 5 6
Clic1 -/-
1 2 3 4 5 6
Clic1 -/- + CLIC1 R29A
1 2 3 4 5 6 other
MCL-1
NC
1 2 3 4 5 6
Clic1 -/- + CLIC1 WT
40kDa
1 2 3 4 5 6
Clic1 -/-
1 2 3 4 5 6
Clic1 -/- + CLIC1 R29A
1 2 3 4 5 6
other
Vinculin
NC
1 2 3 4 5 6
Clic1 -/-
124 kDa
1 2 3 4 5 6
Clic1 -/- + CLIC1 WT
1 2 3 4 5 6
Clic1 -/- + CLIC1 R29A
1 2 3 4 5 6

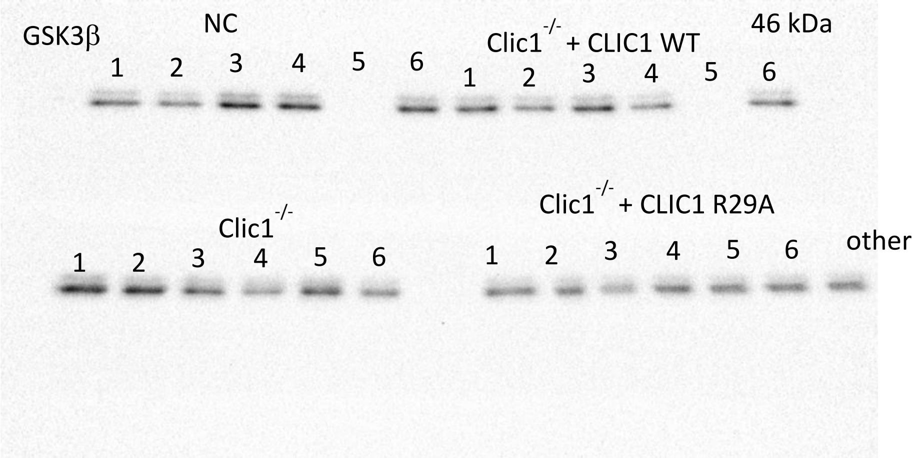
GSK3β
NC
1 2 3 4 5 6
Clic1-/- + CLIC1 WT
1 2 3 4 5 6
46 kDa
Clic1-/-
1 2 3 4 5 6
Clic1-/- + CLIC1 R29A
1 2 3 4 5 6
other

GEL 2

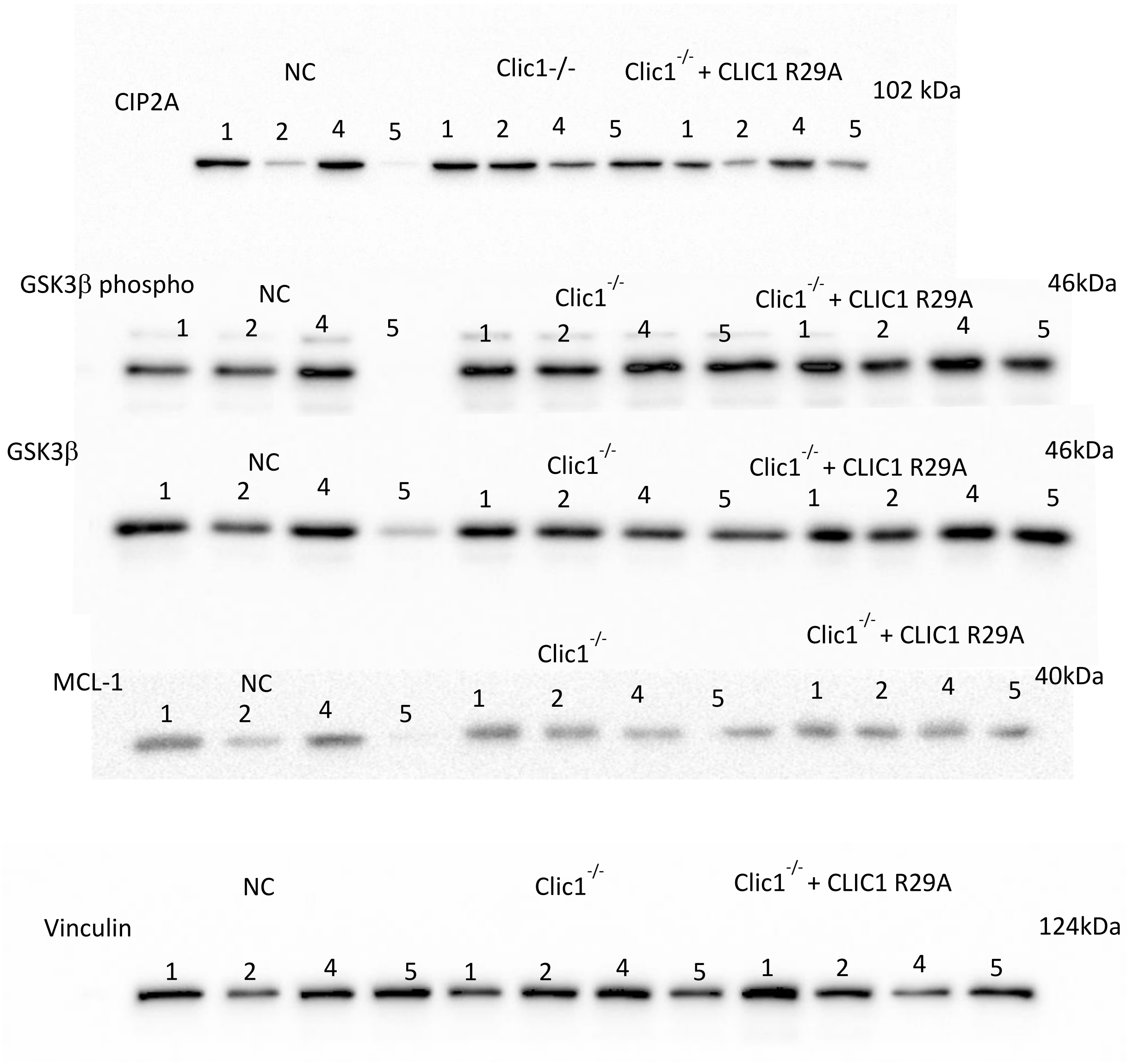
CIP2A
NC
1 2 4 5
Clic1-/-
1 2 4 5
Clic1-/- + CLIC1 R29A
1 2 4 5
102 kDa
GSK3β phospho
NC
1 2 4 5
Clic1-/-
1 2 4 5
Clic1-/- + CLIC1 R29A
1 2 4 5
46kDa
GSK3β
NC
1 2 4 5
Clic1-/-
1 2 4 5
Clic1-/- + CLIC1 R29A
1 2 4 5
46kDa
MCL-1
NC
1 2 4 5
Clic1-/-
1 2 4 5
Clic1-/- + CLIC1 R29A
1 2 4 5
40kDa
Vinculin
NC
1 2 4 5
Clic1-/-
1 2 4 5
Clic1-/- + CLIC1 R29A
1 2 4 5
124kDa

1: High glucose not treated

2: High glucose + 10 mM Metformin

3: High glucose + tmCLIC1omab

4: Low glucose not treated

5: Low glucose + 10 mM Metformin

6: Low glucose + tmCLIC1omab
