# Supplementary material for: Chronic administration of metformin exerts cytostatic and cytotoxic effects via the PP2A-GSK3β-MCL-1 pathway by inhibiting the tmCLIC1 membrane protein in glioblastoma-initiating cells
Source: J Exp Clin Cancer Res. 2025 Nov 24;44:312. doi: 10.1186/s13046-025-03577-3 (PMC12659486; doi:10.1186/s13046-025-03577-3)
Supplement: Supplementary file 2 — Supplementary Material 2. [file 13046_2025_3577_MOESM2_ESM.docx]

**Supplementary Material**


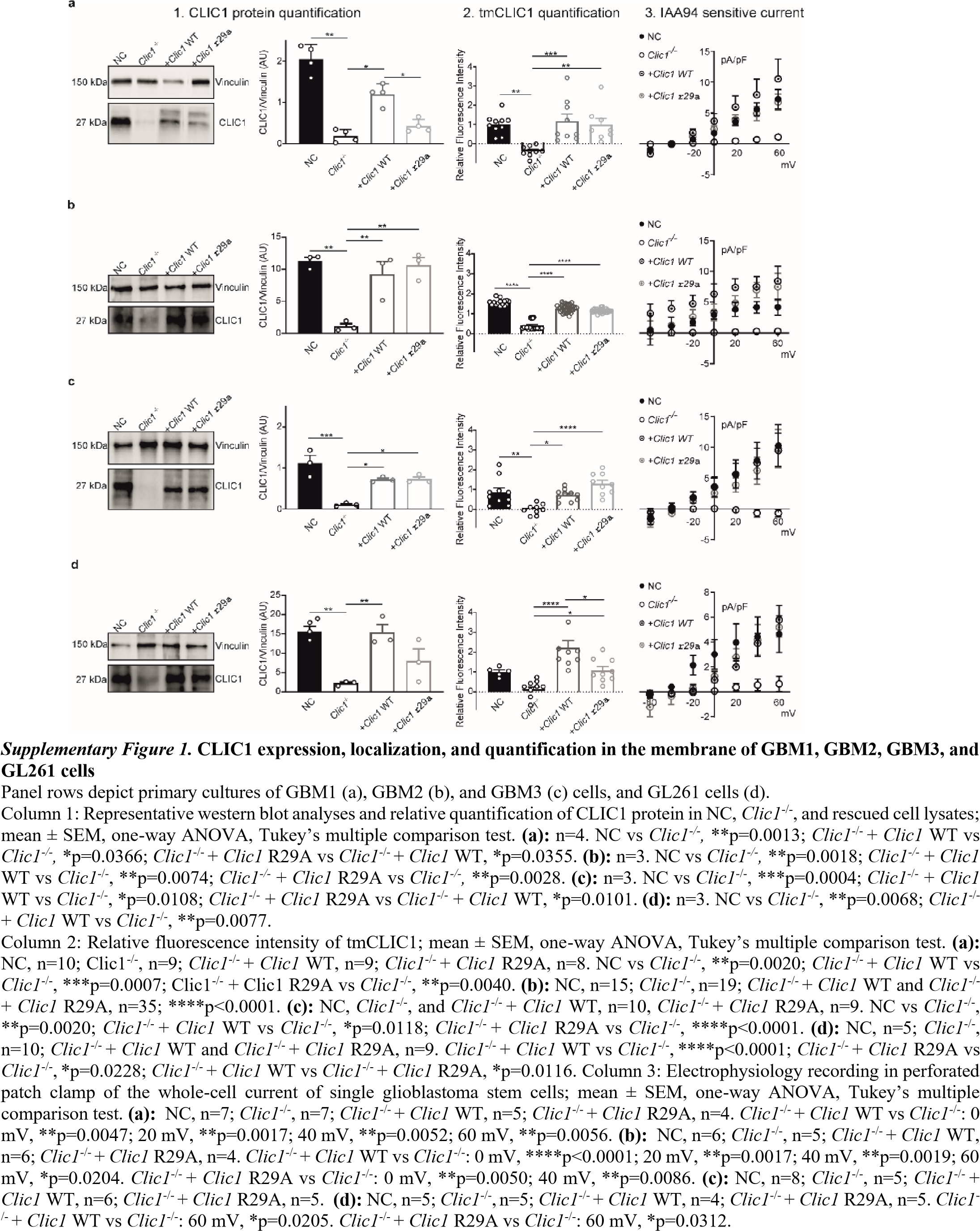


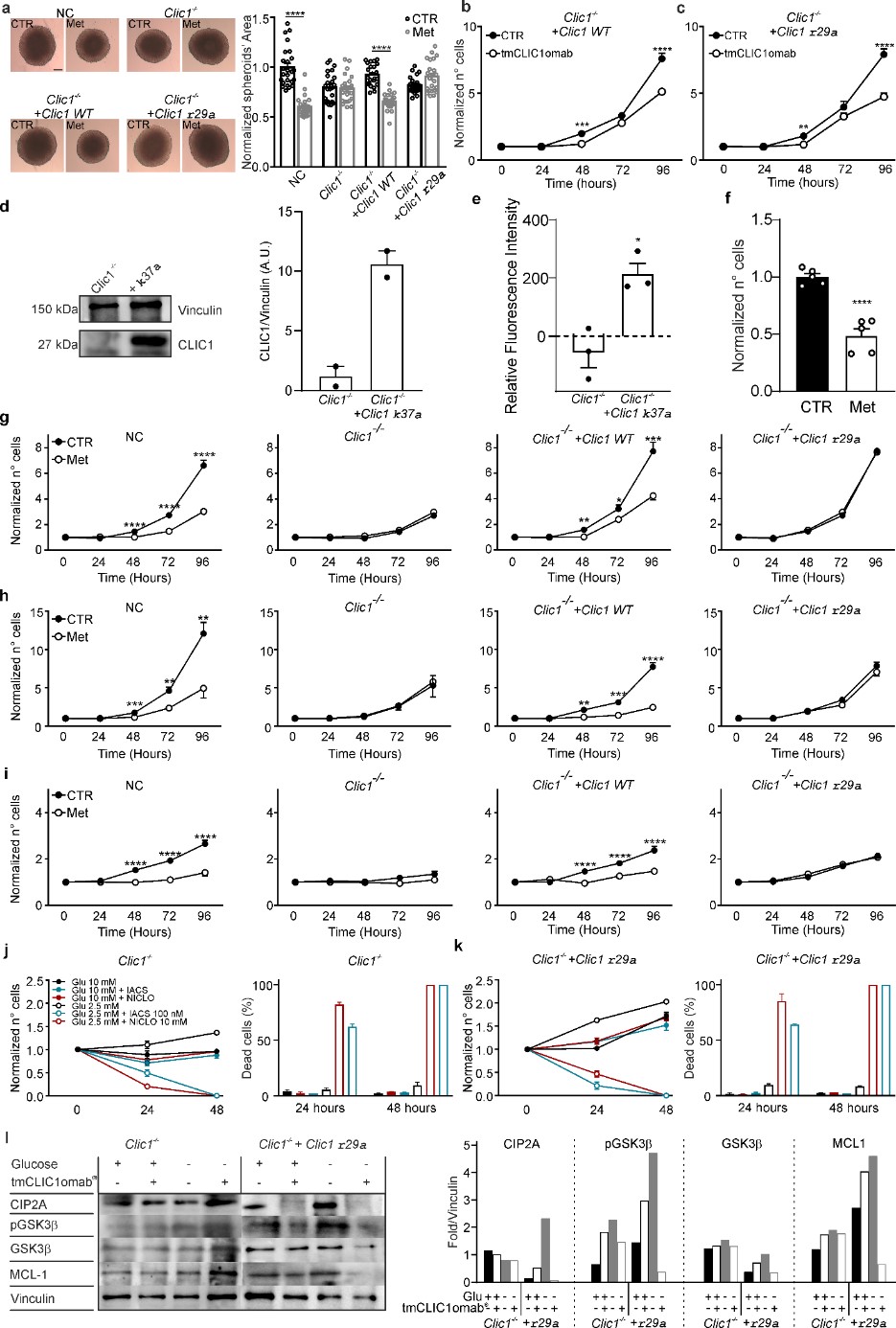


***Supplementary Figure 2 | CLIC1 is responsible for the impairment caused by metformin***

**(a):** (left panel) Representative images showing the development of GBM1 3D structure (spheroid) after 96h of incubation in the absence or presence of 5 mM metformin as indicated in each square (right panel). Quantification of spheroid area in the absence (black circles) or presence (grey circles) of 5 mM metformin. NC : CTR, n=25, Met, n=23 ; ****p<0.0001. *Clic1*^-/-^ : CT, n=25, Met, n=23*. Clic1*^-/-^ + *Clic1* WT: CT, n=23, Met, n=23; ****p<0.0001. *Clic1*^-/-^ + *Clic1* R29A: CT, n=23, Met, n=23. Mean ± SEM, one way ANOVA, Tukey’s multiple comparison test. Scale bar 100 µm. **(b):** Growth curves of GBM3 GSCs over 96h in the absence (black circles) or presence of 3.5 µg/ml tmCLIC1omab antibody (empty circles) in *Clic1*^-/-^ + *Clic1* WT cells. CT, n=8; tmCLIC1omab, n=5; 48h, ***p=0.0003; 96h, ****p<0.0001. **(c):** Growth curves of GBM3 GSCs over 96h in the absence (black circles) or presence of 3.5 µg/ml tmCLIC1omab antibody (empty circles) in *Clic1*^-/-^ + *Clic1* R29A cells. CT, n=8; tmCLIC1omab, n=5; 48h, **p=0.0058; 96h, ****p<0.0001. **(d):** (left) Representative western blot analyses and relative quantification (right) of CLIC1 protein in K37A-rescued GSC lysates. **(e):** Relative fluorescence intensity of tmCLIC1; mean ± SEM, unpaired t-test; *p=0.0127. **(f):** Number of cells after 96h in the absence or presence of 5 mM metformin in K37A-rescued GSCs; mean ± SEM, unpaired t-test; ****P<0.0001. **(g-i):** Growth curves of GBM2 (g), GBM3 (h), and GL261 (i) cells over 96h in the absence (black circles) or presence (empty circles) of 5 mM metformin in NC, *Clic1*^-/-^, *Clic1*^-/-^ + *Clic1* WT, and *Clic1*^-/-^ + *Clic1* R29A. Mean ± SEM, unpaired t-test. (g) NC: CT, 24h, 48h, 96h, n=12, 72h, n=11; Met, 24-48h, n=9, 72-96h, n=8, ****p<0.0001. *Clic1*^-/-^: CT, 24h, 96h, n=11, 48-72h, n=9; Met, 24h, 72h, 96h, n=7, 48h, n=8). *Clic1*^-/-^ + *Clic1* WT: CT, 24-96h, n=10; Met, 24h, 72h, 96h, n=10, 48h, n=9, 48h, **p=0.0015, 72h, *p=0.0185, 96h, ***p=0.0003. *Clic1*^-/-^ + *Clic1* R29A, n=6. (h) NC: 48h, ***p=0.0005, 72h, **p=0.0020, 96h, **p=0.0043. *Clic1*^-/-^ and *Clic1*^-/-^ + *Clic1* R29A, n=6. *Clic1*^-/-^ + *Clic1* WT: CT, n=6; Met, 24-72h, n=6, 96h, n=5, 48h, **p=0.0021, 72h, ***p=0.0002, 96h, ****p<0.0001). (i) NC: CT, 24h, n=9, 48-96h, n=15; Met, 24h, n=9, 48h, 96h, n=15, 72h, n=14), *Clic1*^-/-^: CT and Met, 24h, n=6, 47-96h, n=9. *Clic1*^-/-^ + *Clic1* WT and *Clic1*^-/-^ + *Clic1* R29A, n=12, ****p<0.0001. **(j):** (left) Growth curves of GBM1 GSCs over 48h in high (filled) and low (empty) glucose and in the absence or presence of 100 nM IACS

(light blue) and 10mM niclosamide (dark red) in *Clic1^-/-^* (n=3, 48h IACS and niclosamide, ****p<0.0001). Mean ± SEM, one-way ANOVA, Tukey’s multiple comparison test. (right) Analysis of the percentage of dead cells shown in left panel (n=3; 24-48h IACS and niclosamide, ****p<0.0001); mean ± SEM, one-way ANOVA, Tukey’s multiple comparison test. **(k):** (left) Growth curves of GBM1 GSCs over 48h in high (filled) and low (empty) glucose and in the absence or presence of 100 nM IACS (light blue) and

10mM niclosamide (dark red) in *Clic1^-/-^*+*Clic1* R29A (n=3, 48h IACS and niclosamide, ****p<0.0001). Mean ± SEM, one-way ANOVA, Tukey’s multiple comparison test. (right) Analysis of the percentage of dead cells shown in left panel (n=3; 24-48h IACS and niclosamide, ****p<0.0001); mean ± SEM, one-way ANOVA, Tukey’s multiple comparison test. **(l):** Representative western blot analysis (left) and its quantification (right) of *Clic1^-/-^* and *Clic1^-/-^* + Clic1 R29A cultured in high (black filled column) and low (grey filled column) glucose in the absence (black empty column) or presence (grey empty column) of 3.5 µg/ml of tmCLIC1omab^®^.


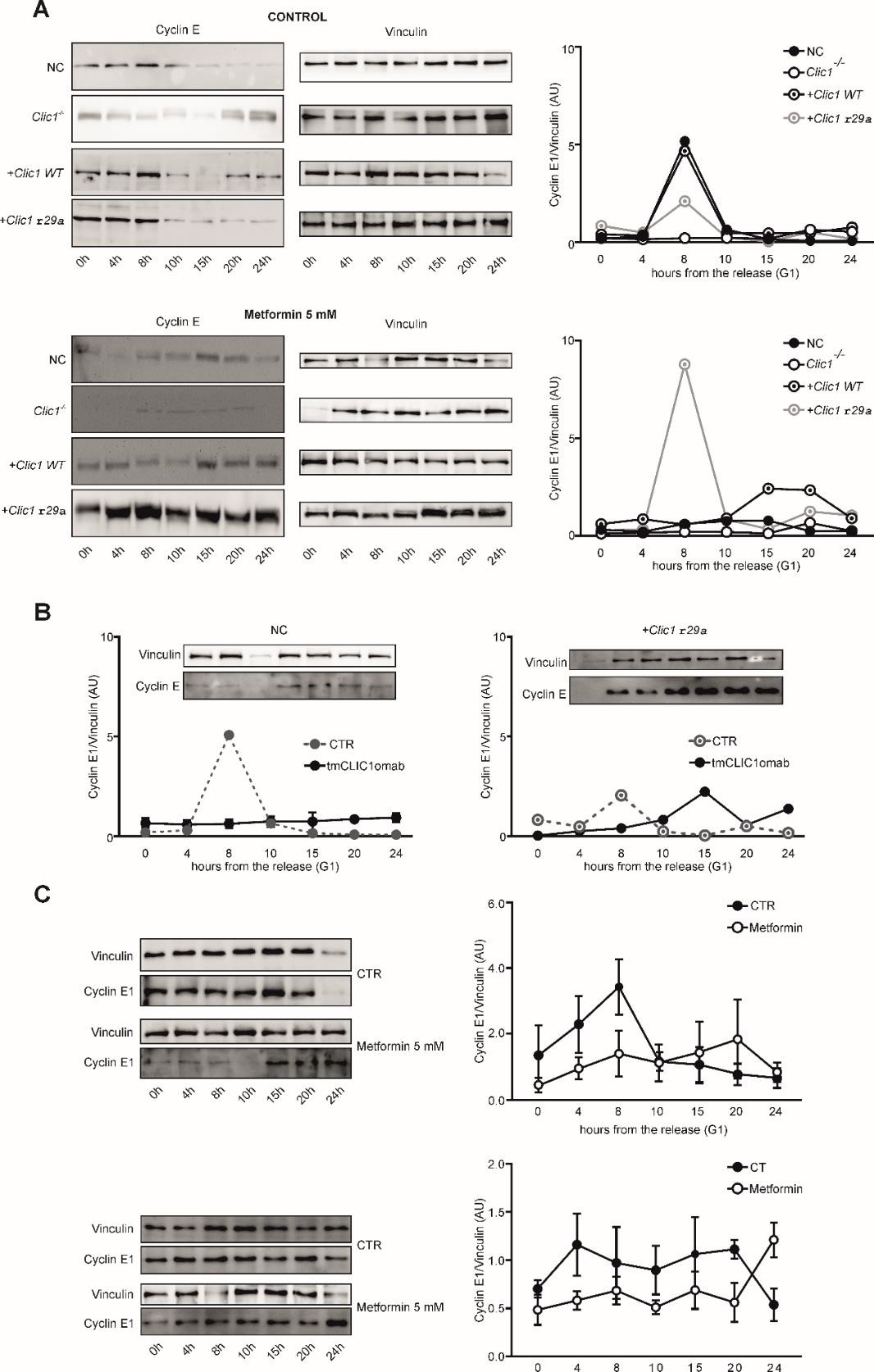
 **Supplementary Figure 3 | Cyclin E expression in**

**GBM1, GBM2, and**

**GBM3 primary cultures**   **(a):** (left) Representative western blot analyses for Cyclin E1 expression in lysates of NC, *Clic1*^-/-^, and rescued GBM1 GSC populations over time after the release from G1 synchronization, in absence (top) or presence

(bottom) of 5mM metformin. (right) Trend of Cyclin E1 protein expression in the samples

(n=3).

**(b):** (top) Representative western blot analyses for Cyclin E1 expression in lysates of NC (left) and *Clic1*^-/-^ + *Clic1* R29A (right) GBM1 populations over time after the release from G1 synchronization, in the presence of 3.5 µg/ml tmCLIC1omab.


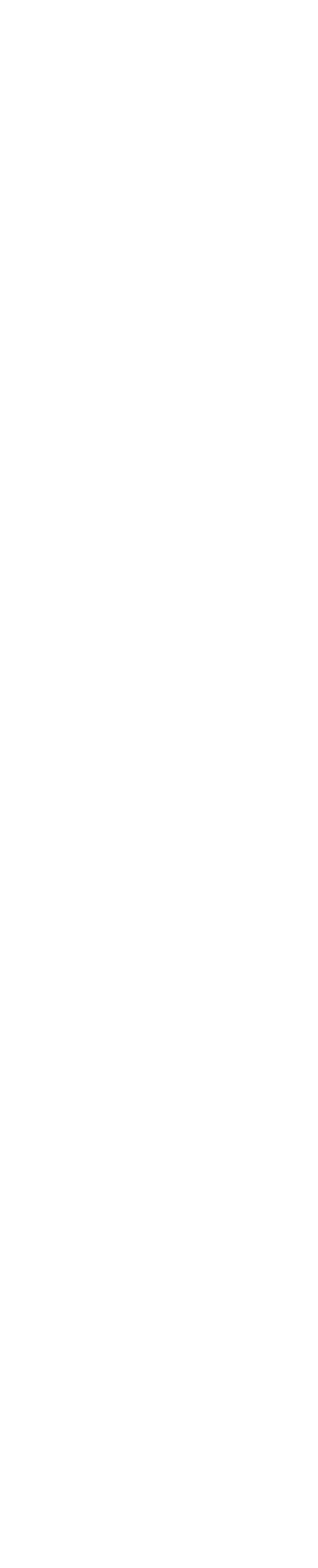
 (bottom) Trend of Cyclin E1 protein expression in the treated samples (filled circles; n=3). Dashed lines indicate the untreated samples shown in (a).  **(c):** (left) Representative western blot analyses for Cyclin E1 expression in lysates of NC GBM2 (top) and GBM3 (bottom) cell populations over time after the release from G1 synchronization, in absence or presence of 5mM metformin. (right) Trend of Cyclin E1 protein expression (n=3) in control (black circles) and metformin-treated cells (empty circles).


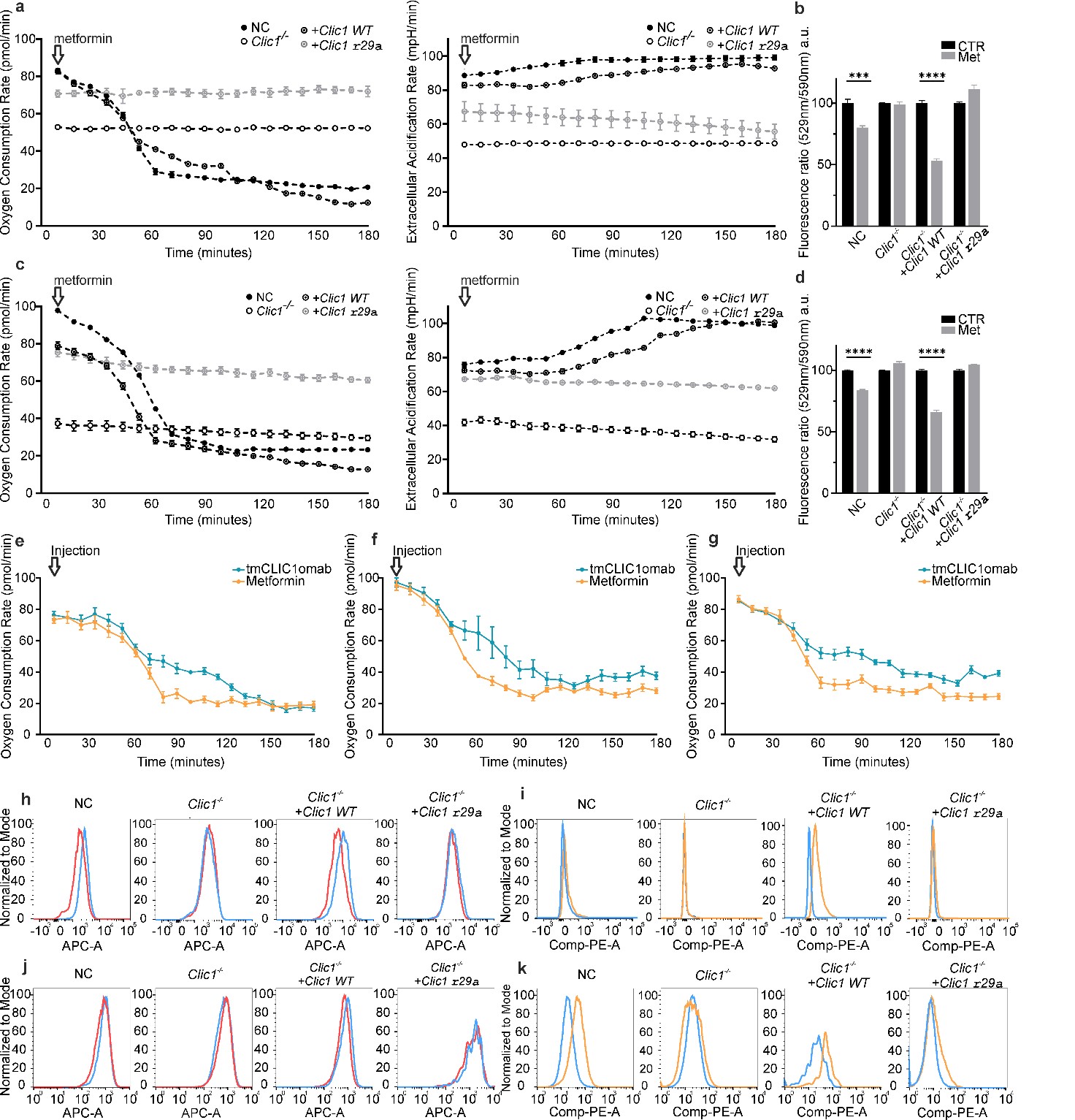


# Supplementary Figure 4 | Metformin’s effect on metabolism depends on CLIC1


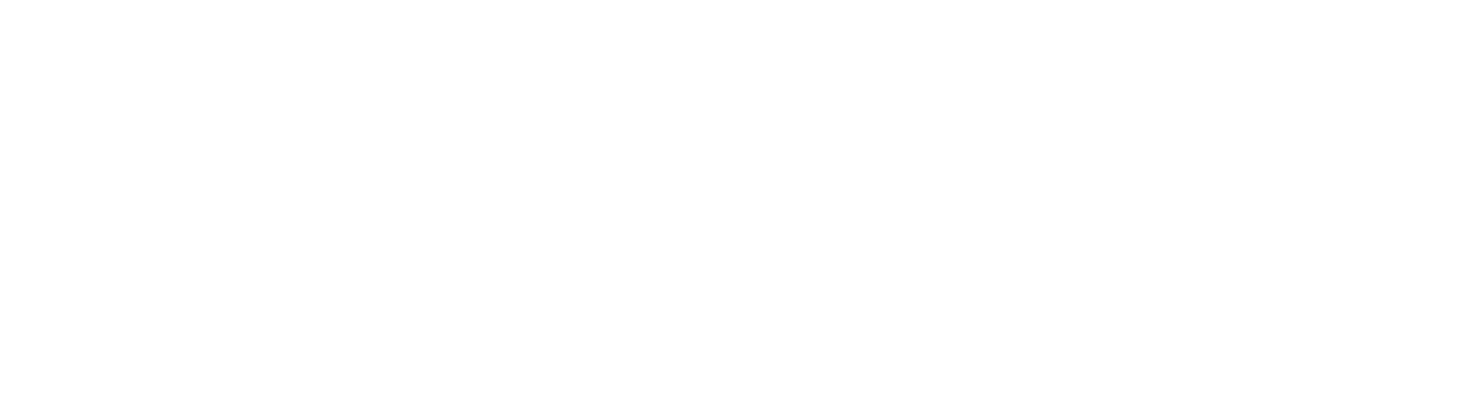
 **(a and c):** Oxygen consumption rate (left) and extracellular acidification rate (right) in GBM2 (a) and GBM3 (c) as an indicator of oxidative phosphorylation and glycolytic activity after acute injection of 10 mM metformin in NC (black circles), *Clic1^-/-^* (empty circles), *Clic1^-/-^* + Clic1 WT (black circle dot), *Clic1^-/-^* + Clic1 R29A cells (grey circle dot). Each experimental point was taken every 6 min. **(b and d):** Mitochondrial membrane potential in GBM2 (b) and GBM3 (d) measured through a JC-1 probe in NC, *Clic1^-/-^*, *Clic1^-/-^* + *Clic1* WT, and *Clic1^-/-^* + *Clic1* R29A in the absence (black columns) or presence (grey columns) of 5 mM metformin. n=2. GBM2, NC: CT vs Met, **p=0.0008. *Clic1*^-/-^+ *Clic1* WT, CT vs Met, ****p<0.0001. GBM3, ****p<0.0001. Mean ± SEM, two-way ANOVA, Sidak’s multiple comparison test. **(e-g):** Oxygen consumption rate in GBM1 (e), GBM2 (f), and GBM3 (g) after acute injection of 10 mM metformin (orange) and 3.5 µg/ml tmCLIC1omab antibody (cyan). **(h and j):** Representative plot of FACS analysis of CellROX^TM^ Deep Red Reagent fluorescence for oxidative stress detection in GBM2 (h) and GBM1 (j). Cells were incubated for 3h in the absence (blue) or presence (red) of 5mM of metformin. **(i and k):** Representative plot of FACS analysis of MitoSOX^TM^ Red Indicator fluorescence for mitochondrial superoxide detection in GBM2 (i) and GBM1 (k). Cells were incubated for 3h in the absence (blue) or presence (orange) of 5mM metformin.


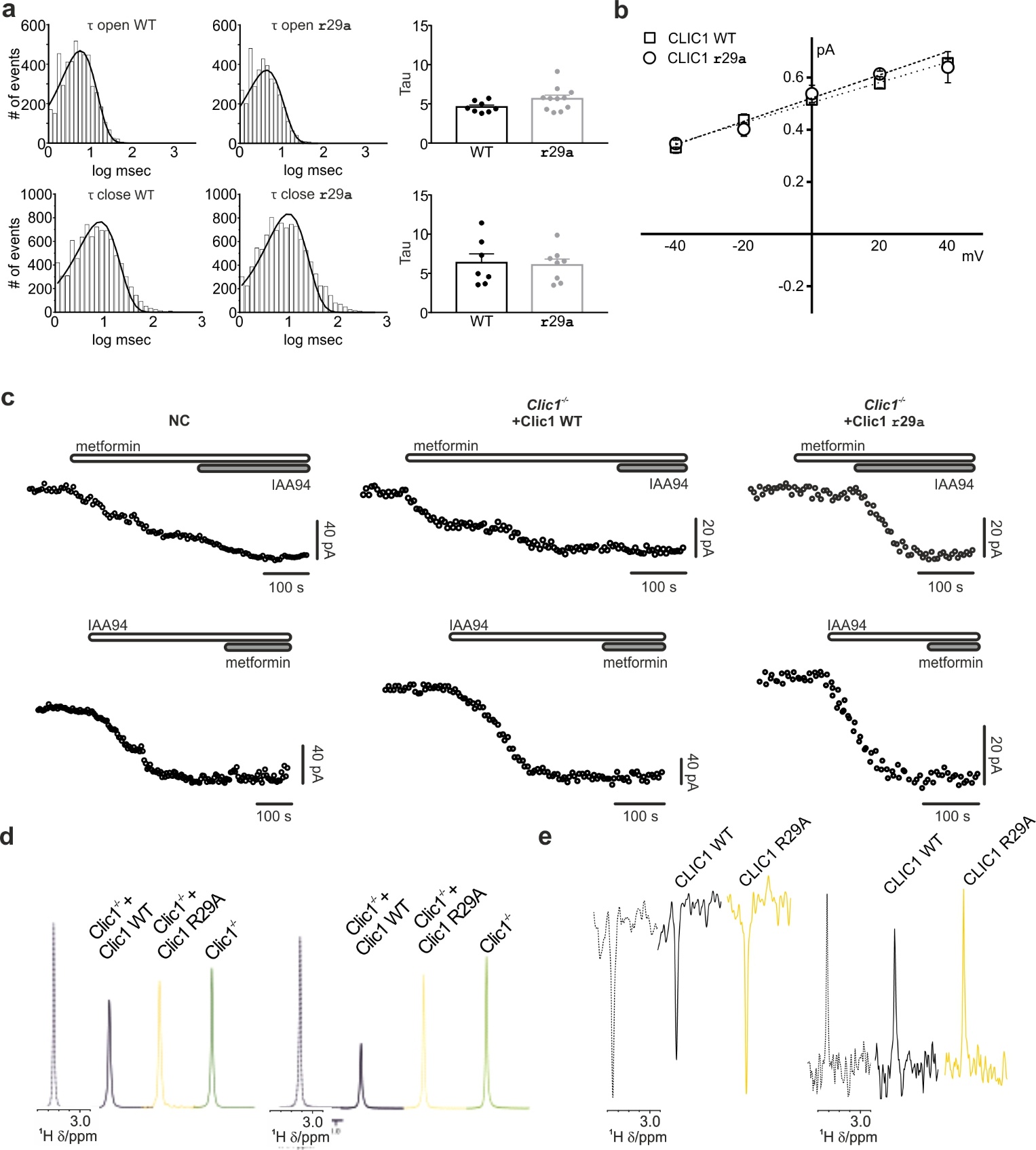


**Supplementary Figure 5 | Metformin directly interacts with tmCLIC1**

**a:** (left) Open and close times (t) of tmCLIC1 channels in *Clic1*^-/-^ + *Clic1* WT and *Clic1*^-/-^ + *Clic1* R29A GBM3 cells measured for at least 3 min of continuous recording in outside-out configuration. Histograms were best fitted by a single exponential decay function (bold line). (right) Quantification of single τ values. τ close: *Clic1*^-/-^ + *Clic1* WT, 4.59 ms ± 0.22 (n=9); *Clic1*^-/-^ + *Clic1* R29A, 5.65 ms ± 0.46 (n=11). τ close: *Clic1*^-/-^ + *Clic1* WT, 6.35 ms ± 1.13 (n=7); *Clic1*^-/-^ + *Clic1* R29A, 6.06 ± 0.75 ms (n=8). **b:** Current-voltage relationship of tmCLIC1 single-channel outside-out experiments. The calculated conductance was 3.89 pS ± 0.21 for *Clic1*^-/-^ + *Clic1* WT and 4.46 pS ± 0.27 for *Clic1*^-/-^ + *Clic1* R29A. **c:** Representative time-course of whole-cell currents in NC and WT/R29A rescued cells. Each point represents the average current of the last 100 ms of a single current trace. Cells were stimulated every 5 s with an 800 ms, +60 mV test potential from the resting potential. Once the current amplitude reached a constant value, 5 mM metformin then 100µM IAA94 were perfused (top), or in reverse order (bottom). **d:**T_2_ filter ^1^H NMR spectra of 2 mM metformin in the absence (dashed line) or presence of Clic1-/- (green), Clic1-/- + Clic1 WT (black), and Clic1-/- + Clic1 R29A (yellow) cells, recorded using the standard (right), and double cell number (left). .**e:** WaterLOGSY and T_2_ filter ^1^H NMR spectra of 50 µM metformin in the absence (dashed line) and presence of 10 µM CLIC1 WT (black) or R29A mutant (yellow). Only the spectral region containing the metformin dimethyl resonance signal is shown. Arrows highlight signal changes, showing metformin binding to CLIC1 WT; no changes are observed in the presence of the R29A mutant.


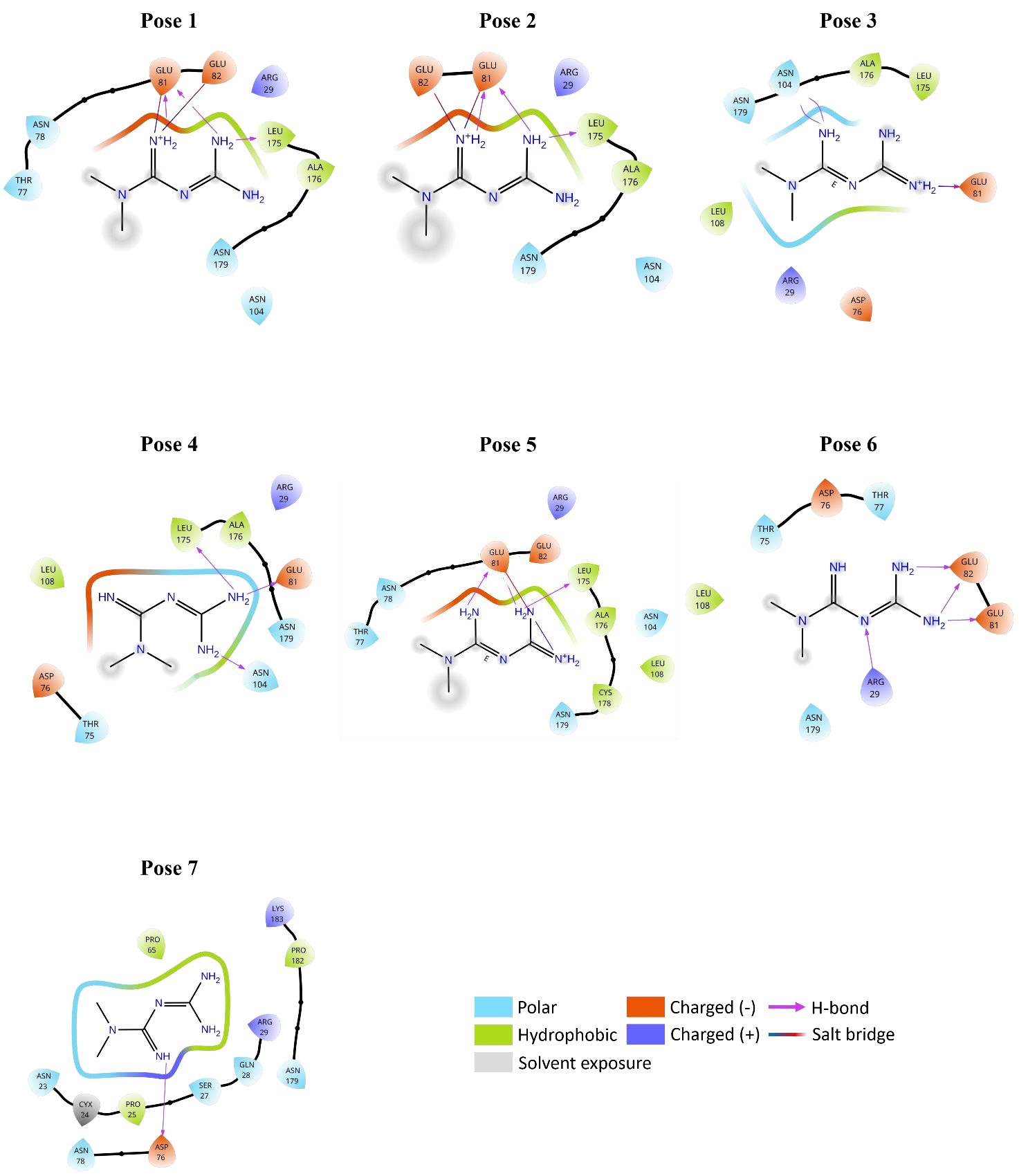


**Supplementary Figure 6 | Bidimensional protein-ligand interaction diagrams.**

The best docking poses of metformin targeting CLIC1 are represented as 2D profiles, with the protein-ligand interactions shown within a cutoff distance of 3 Å.

| **Residues** | **Pose 1** -2.141 | **Pose 2** -2.139 | **Pose 3** -1.864 | **Pose 4** -1.623 | **Pose 5** -1.446 | **Pose 6** -1.360 | **Pose 7** -1.191 |
| --- | --- | --- | --- | --- | --- | --- | --- |
| R29 |  |  |  |  |  | h-bond |  |
| D76 |  |  |  |  |  |  | h-bond |
| E81 | h-bond salt bridge | h-bond salt bridge | salt bridge | h-bond | h-bond salt bridge | h-bond |  |
| E82 | salt bridge | salt bridge |  |  |  | h-bond |  |
| N104 |  |  | h-bond | h-bond |  |  |  |
| L175 | h-bond | h-bond |  | h-bond | h-bond |  |  |

**Supplementary Table 1| CLIC1-metformin interactions in docking poses.**

Table summarizing the main interactions between metformin and residues within the CLIC1 binding pocket across the seven analyzed docking poses. Docking scores are shown for each pose. Specific interactions, including hydrogen bonds and salt bridges, are listed for residues arginine 29, aspartate 76, glutamate 81, glutamate 82, asparagine 104, and leucine 175.


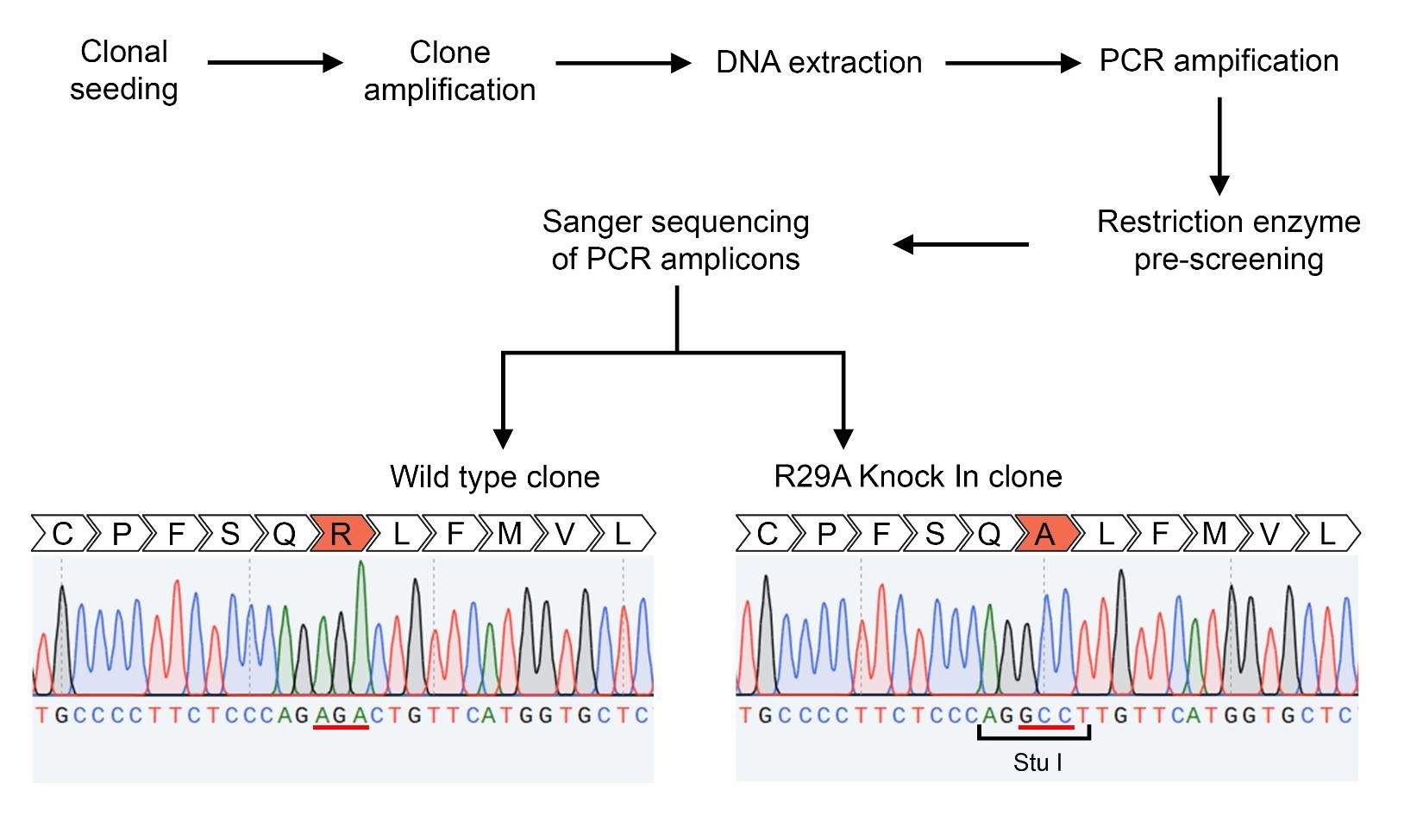


**Supplementary Figure 7:** **Schematic representation of the workflow for screening the R29A edited GL261 clones.** Sequencing electropherogram of PCR amplicons of wild type and R29A Knock In clones confirms the R29A substitution. The red bars indicate the wild type AGA and the mutated GCC triplet coding for arginine and alanine, respectively. The StuI site is highlighted (black bar).
